# TBL2 Promotes Tumorigenesis via PRMT5/WDR77‐Mediated AKT Activation in Breast Cancer

**DOI:** 10.1002/advs.202400160

**Published:** 2024-11-05

**Authors:** Xiuqing Lu, Chao Zhang, Lewei Zhu, Sifen Wang, Lijun Zeng, Wenjing Zhong, Xuxia Wu, Qi Yuan, Hailin Tang, Shien Cui, Yeru Tan, Yuehua Li, Weidong Wei

**Affiliations:** ^1^ State Key Laboratory of Oncology in South China Guangdong Provincial Clinical Research Center for Cancer Sun Yat‐Sen University Cancer Center Guangzhou Guangdong 510000 China; ^2^ District 2 Breast Center Zhongshan City People's Hospital Zhongshan 528400 China; ^3^ Department of Breast Surgery The First People's Hospital of Foshan Foshan Guangdong 528000 China; ^4^ The First Affiliated Hospital Hengyang Medical School University of South China Hengyang Hunan 421001 China

**Keywords:** AKT phosphorylation, breast cancer, PRMT5/WDR77, proliferation, TBL2

## Abstract

Breast cancer (BC) is a common malignancy that affects women worldwide. Although transducing beta‐like 2 (TBL2), a member of the WD40 repeat protein family, has been implicated in various intracellular signaling pathways, its precise function in BC remains unclear. The expression of TBL2 is analyzed using real‐time PCR, western blotting, and immunohistochemistry in BC patient specimens. Kaplan–Meier survival analysis is employed to assess its prognostic significance. Proteomic analysis, immunoprecipitation tests, and protein immunoblotting are employed to examine the impact of TBL2 on AKT phosphorylation activation. The findings reveal selective overexpression of TBL2 in BC, correlating significantly with various clinicopathological characteristics and poor survival outcomes in patients with BC. Through in vivo and in vitro experiments, it is observed that TBL2 suppression inhibits BC cell proliferation, while TBL2 overexpression has the opposite effect. Mechanistically, TBL2 is identified as a scaffolding protein that promotes PRMT5 and WDR77 interaction. This interaction enhances the methyltransferase activity of PRMT5, leading to increased AKT phosphorylation activation and promotion of breast cancer cell proliferation. In conclusion, this study uncovers a novel function of TBL2 in the activation of AKT by PRMT5 and suggests TBL2 as a potential therapeutic target for BC treatment.

## Graphical abstract

TBL2 plays a crucial role in breast cancer progression. Through comprehensive analysis of patient samples, the study reveals TBL2's selective overexpression in breast cancer, correlating with poor prognosis. Mechanistically, TBL2 acts as a scaffold protein enhancing PRMT5‐WDR77 interaction, leading to increased AKT activation and breast cancer proliferation. This highlights TBL2 as a promising therapeutic target for breast cancer treatment.

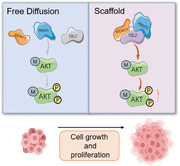

## 1 Introduction

Breast cancer remains a major global health concern, characterized by elevated incidence and mortality rates in numerous regions.^[^ ^1^ ^]^ Despite progress in treatment development,^[^ ^2^, ^3^, ^4^, ^5^ ^]^ the identification of novel biological indicators and therapeutic targets is crucial for advancing personalized and effective approaches to BC management.^[^ ^6^, ^7^ ^]^

Protein kinase B (PKB), is a critical regulator of cell growth and differentiation in the PI3K/AKT/mTOR signaling pathway.^[^ ^8^, ^9^ ^]^ Dysregulation of AKT has been implicated in various human cancers, including breast, ovarian epithelial, prostate, and gastric cancers.^[^ ^10^, ^11^, ^12^ ^]^ AKT activation is tightly controlled through multiple steps, involving PI3K‐mediated conversion of PIP2 to PIP3 as the primary mechanism, followed by phosphorylation at Thr308 and Ser473 by PDK1 and mTORC2, respectively.^[^ ^13^, ^14^, ^15^ ^]^ Additionally, post‐translational modifications (PTMs), such as methylation and glycosylation, have been shown to modulate AKT activation.^[^ ^13^ ^]^ Recent studies highlight the essential role of PRMT5 (protein arginine methyltransferase 5), a type II protein arginine methyltransferase, in conjunction with its partner WDR77, in mediating AKT methylation, phosphorylation and activation, thereby facilitating various processes associated with tumor growth and progression.^[^ ^16^, ^17^ ^]^

Transducin beta‐like 2 (TBL2), a member of the WD40 repeat protein family, is involved in regulating intracellular signaling pathways, including TGF, PERK, and PI3K‐AKT signaling pathways.^[^ ^18^, ^19^, ^20^ ^]^ Emerging research suggests that TBL2, acting as a driver gene, enhances endoplasmic reticulum stress and promotes the proliferation of lung adenocarcinoma cells.^[^ ^21^ ^]^ Abnormal TBL2 regulation has been linked to various diseases such as Williams‐Beuren syndrome, dyslipidemia, and bladder cancer,^[^ ^22^ ^]^ while its exploration in BC remains limited. Moreover, the precise molecular function of TBL2 remains unknown, necessitating further research to comprehensively elucidate and establish its potential as a therapeutic target for BC.

In this study, we observed significant upregulation of TBL2 in BC and identified TBL2 as a predictor of poor prognosis in BC patients. Furthermore, we elucidated that TBL2 performs a critical supporting role, facilitating effective interaction between PRMT5 and WDR77, consequently promoting AKT phosphorylation and activation. Our findings demonstrate the critical involvement of TBL2 in PRMT5‐mediated growth and proliferation in BC. These novel insights into the role of TBL2 in AKT activation and BC growth offer potential therapeutic opportunities by targeting TBL2.

## 2 Results

### 2.1 TBL2 is Upregulated in Human Breast Cancer

**Figure 1:**
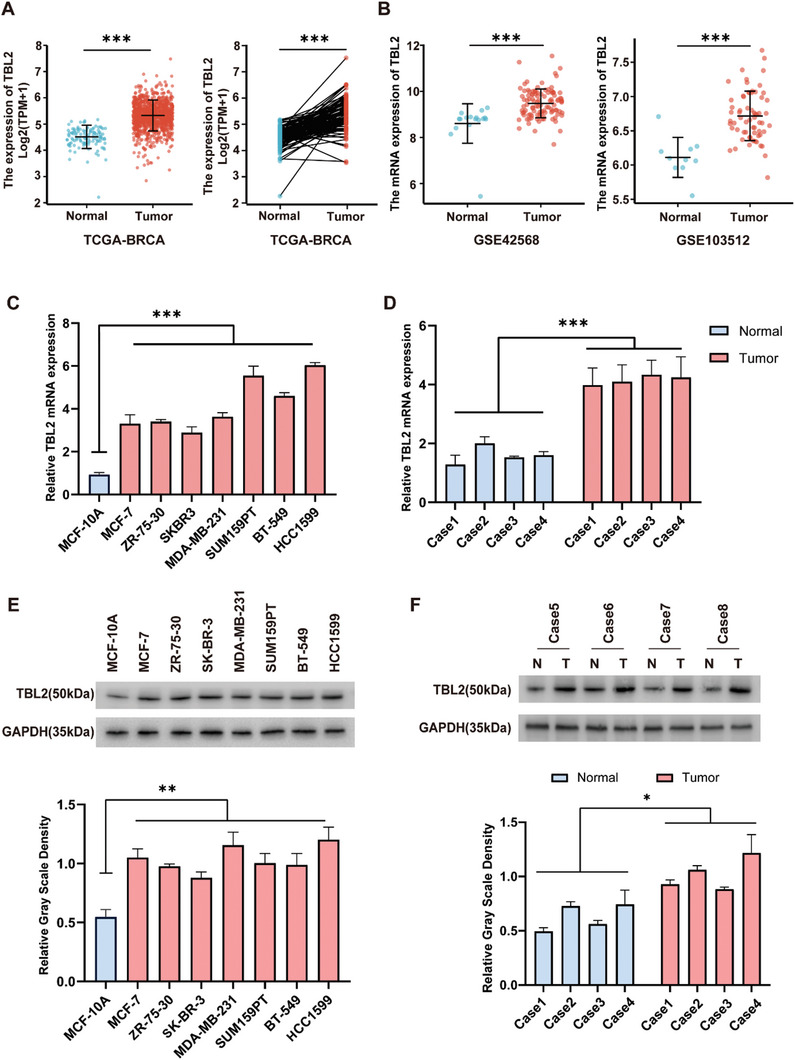
TBL2 is upregulated in Human breast cancer. A) Comparison of TBL2 mRNA expression levels between tumor and normal tissues in the TCGA breast cancer dataset, including unpaired samples (left panel) and paired samples (right panel) (113 normal and 1095 tumor samples). B) Comparison of TBL2 mRNA expression levels between tumor and normal tissues in the GEO datasets (left panel: GSE42568, including 17 normal, 104 BC samples; right panel: GSE103512, including 10 normal, 65 BC samples). C,E) qRT‐PCR C) and western blotting E) analyses of TBL2 expression in human breast cancer cell lines. D,F) qRT‐PCR D) and western blotting F) analyses of TBL2 expression in BC tissues and adjacent normal tissue. GAPDH was used as a loading control. Data represent the means ± S.D. of three independent experiments. ^*^ *P* < 0.05; ^**^ *P *< 0.01; ^***^ *P* < 0.001.

We first examined the expression of TBL2 in human breast cancer tissues using the Cancer Genome Atlas dataset (TCGA), which revealed an upregulation compared to normal breast tissues (**Figure** **1**A). A similar trend was observed in Gene Expression Omnibus (GEO) datasets (Figure 1B). To validate these observations, we conducted real‐time PCR and western blotting analyses, which consistently exhibited a significant increase in TBL2 expression in both BC tumor tissues and cell lines (Figures 1C–F). These data provide compelling evidence supporting the specific upregulation of TBL2 in human breast cancer tissues.

### 2.2 High TBL2 Expression Indicates Poor Prognosis

**Figure 2:**
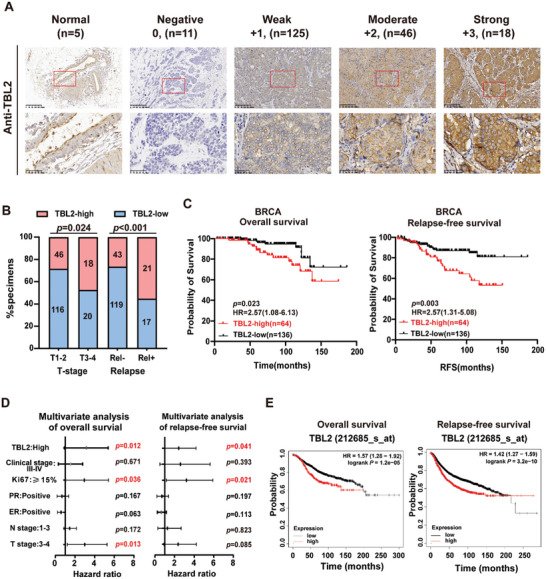
High TBL2 expression indicates poor prognosis. A) Representative images of TBL2 IHC staining scored as negative (0), weak (+ 1), moderate (+ 2), or strong (+ 3). The number of samples corresponding to each score is shown in brackets. B) Analysis of the correlation between TBL2 staining, T stage, and relapse state in patients with BC. The χ2 test was used for analysis. C) Kaplan–Meier OS and RFS curve for patients with BC stratified by low and high TBL2 expression (log‐rank test). D) The significance of the relationship between the TBL2 signature and OS and RFS in the presence of additional clinical factors, as assessed using multivariate Cox regression analysis. E) All breast cancer patients’ OS and RFS were analyzed using the Kaplan–Meier Plotter (http://kmplot.com/analysis) software. Except for gene symbol (TBL2), survival (OS and RFS), and auto‐select best cutoff (on), all settings were kept at their default levels. HR, hazard ratio; OS, overall survival; RFS, recurrence‐free survival.

We collected and analyzed clinical follow‐up data and pathological specimens from 200 patients with invasive ductal carcinoma at the Sun Yat‐sen University Cancer Center (Table S2, Supporting Information). We performed an IHC analysis of TBL2 expression and found cytoplasmic positivity for TBL2 in 94.5% of the samples, with distinct staining around the nucleus. Additionally, TBL2 was expressed more prominently in BC tissues than in adjacent normal tissues (**Figure** **2**A). Correlation analysis between TBL2 expression levels and clinical pathological staging revealed that patients in the high TBL2 expression group had a later T‐stage (*P* = 0.024) and a higher tumor local recurrence rate (Local recurrence defined as a relapse on either chest wall or in a regional lymph node or distant recurrence) (*P* < 0.001) compared to those in the low TBL2 expression group (Figure 2B). Log‐rank survival analysis demonstrated that patients with low TBL2 expression exhibited longer RFS (recurrence‐free survival, defined as the time interval between surgery and the first recurrence) (*P* = 0.003) and OS (overall survival, defined as the time from diagnosis to death from any cause) (*P* = 0.023) than those with high TBL2 expression (Figure 2C). Furthermore, multivariate proportional hazards regression models indicated TBL2 expression as an independent influencing factor for RFS (*P* = 0.041) and OS (*P* = 0.012) (Figure 2D). According to Kaplan–Meier Plotter, an online database (http://kmplot.com/analysis), all BC patients with high TBL2 expression had shorter RFS and OS (Figure 2E). These results suggest that TBL2 may accelerate the malignant progression of BC.

### 2.3 TBL2 Promotes Breast Cancer Cell Proliferation

**Figure 3:**
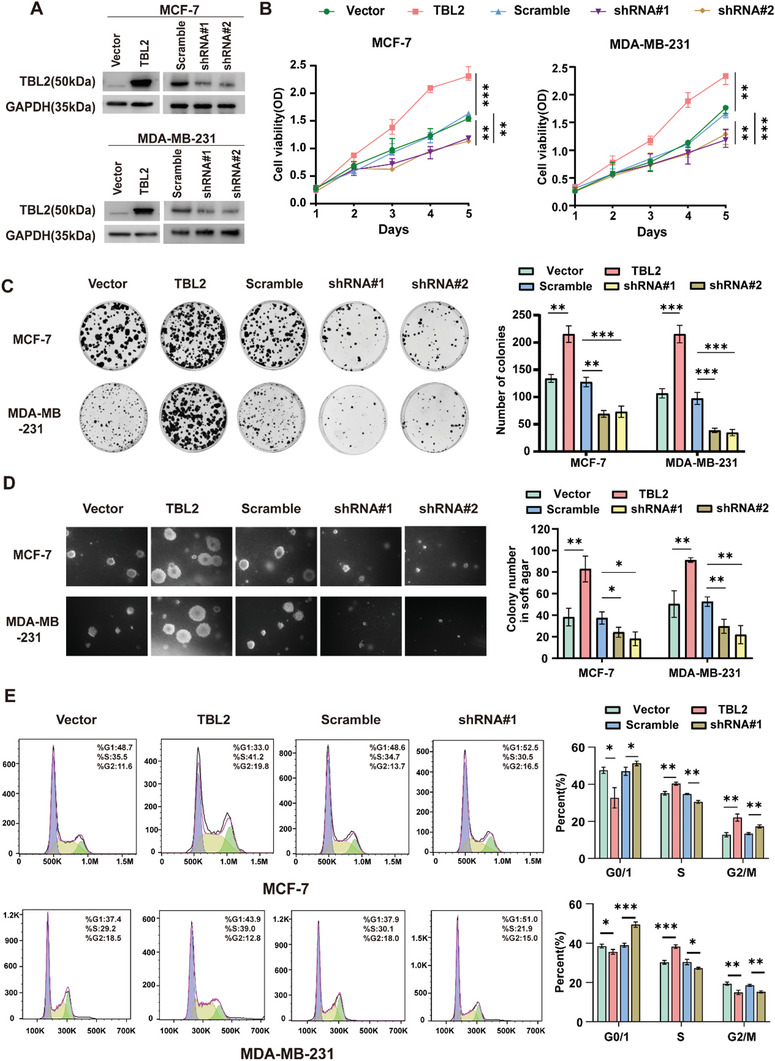
TBL2 promotes breast cancer cell proliferation. A) Western blotting analysis of TBL2 in MCF‐7 and MDA‐MB‐231 cells stably transfected with TBL2‐overexpressed and TBL2‐silenced plasmids. GAPDH was utilized as a control for protein loading. B–E) Experiments were conducted in the indicated cells using CCK‐8 B), colony formation C), anchorage‐independent growth D), and flow cytometric E) assays. A two‐tailed Student's t‐test was used. Data represent the means ± S.D. of three independent experiments. ^*^ *P* < 0.05; ^**^ *P *< 0.01; ^***^ *P* < 0.001.

We examined the role of TBL2 in the progression of breast cancer through gain or loss‐of‐function methods. Two human BC cell lines, MCF‐7 and MDA‐MB‐231, were genetically modified to either overexpress or silence TBL2 (**Figure** **3**A). TBL2 overexpression in BC cells increased cell proliferation, colony formation, anchorage‐independent growth, and cell‐cycle transition, while TBL2 depletion had the opposite effect (Figures 3B–E). Moreover, to further investigate the impact of TBL2 knockdown on cell growth, we performed a gradient transfection of MDA‐MB‐231 cells with TBL2siRNA followed by a CCK8 assay. We observed that cells transfected with 100nM of siRNA exhibited weaker growth ability, which was statistically different from those transfected with only 30nM (Figures S1A,B, Supporting Information). Similar results were observed in the mouse‐derived BC cell line, 4T1, suggesting that TBL2 plays a conserved role in promoting BC progression (Figures S1C–F, Supporting Information). However, TBL2 did not significantly affect the invasion and metastasis of BC cells (Figures S1G,H, Supporting Information).

**Figure 4:**
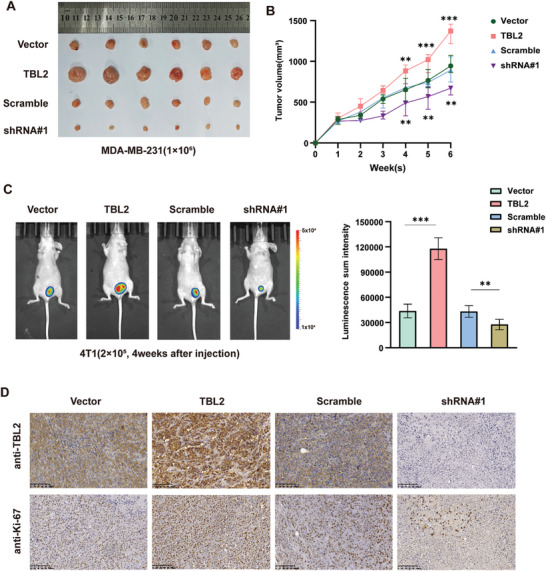
TBL2 facilitates breast cancer tumor growth. A) Control, TBL2‐overexpressed, or TBL2‐silenced MDA‐MB‐231 cells were orthotopically injected into nude mice (1 × 10^6^/injection, n = 6/group). B) Comparison of tumor volumes in various groups (n = 6). C) The tumor burden of different groups is determined by the luciferase signal (n = 6). D) IHC results of Ki‐67 staining in the specified xenografts. Statistical analysis was conducted using a two‐tailed Student's t‐test. ^*^ *P* < 0.05; ^**^ *P* < 0.01; ^***^ *P* < 0.001.

The tumor‐promoting effects of TBL2 on cell proliferation were further verified in in vivo studies using orthotopic mouse models of BC. Tumor growth was significantly accelerated in the TBL2‐overexpressing group and suppressed in the TBL2‐silenced group (**Figures** **4**A,B; Figure S1I, Supporting Information). To confirm the impact of TBL2 on proliferation, we utilized 4T1 cells to establish an orthotopic mouse model of BC. The mammary fat pad was orthotopically injected with luciferase‐expressing 4T1 cells (2 × 10^5^) that had TBL2 expression modified. Weekly BLI TBL2 Acts as a Scaffold to Promote the Interaction Between PRMT5 and WDR77(Bioluminescence imaging) showed higher tumor loads in the TBL2‐overexpression group and lower tumor loads in the TBL2‐silenced group (**Figure** **4**C). Moreover, elevated Ki‐67 values in tumors with TBL2 overexpression suggested increased proliferation (Figure 4D). These results indicate the critical role of TBL2 in promoting breast cancer progression.

### 2.4 TBL2 Promotes AKT Phosphorylation and Activation

**Figure 5:**
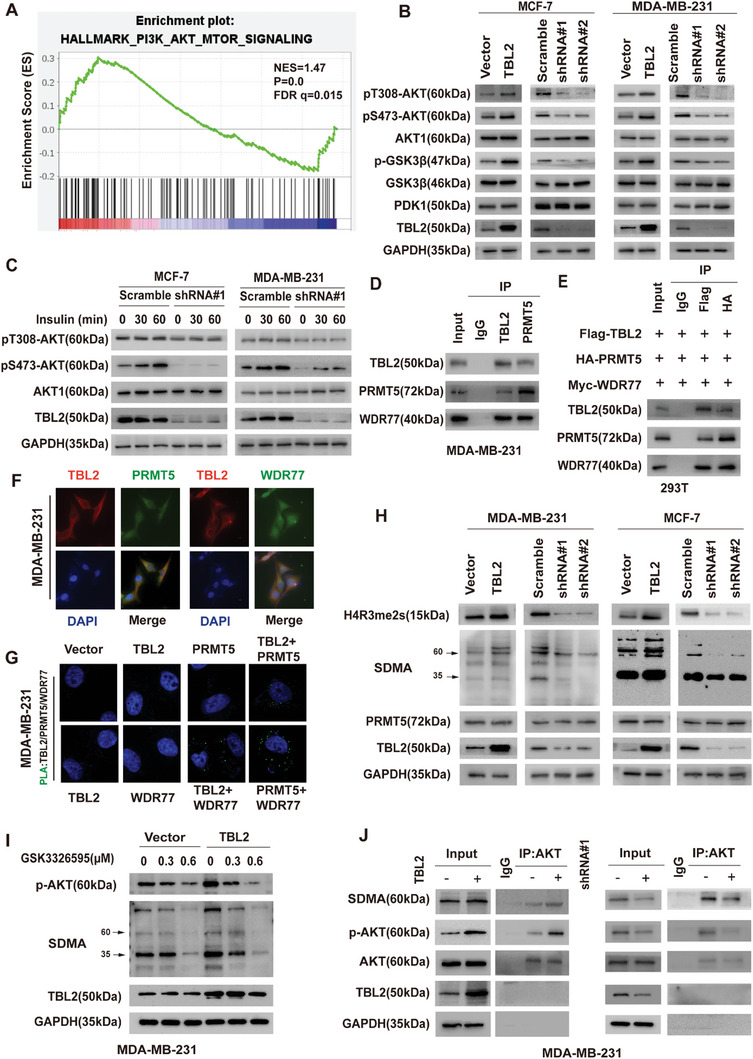
TBL2 promotes AKT phosphorylation and interacts with PRMT5 and WDR77. A) The TCGA breast cancer dataset was subjected to GSEA after stratification based on TBL2 expression. Enrichment of HALLMARK_PI3K_AKT_MTOR_SIGNALING was indicated. FDR q, false discovery rate q value; NES, normalized enrichment score. B) Western blotting analysis of TBL2, p‐AKT (Thr308), p‐AKT (Ser437), AKT1, p‐GSK3β, GSK3β, and PDK1 in TBL2‐transduced cells and TBL2‐silenced cells compared with control cells. GAPDH was used as a control for protein loading. C) Western blotting analysis of the AKT phosphorylation levels. Cells were subjected to serum starvation for 16 h and treated with 100 nM insulin for 0, 30, and 60 min before collection. D)IP assays determined the binding between endogenous TBL2, PRMT5, and WDR77 in MDA‐MB‐231 cells. E) IP assays were performed in 293T cells transfected with Flag‐TBL2, HA‐PRMT5, and MYC‐WDR77 constructs. F) Immunofluorescence assay demonstrating that TBL2, PRMT5, and WDR77 were at least partially colocalized in MDA‐MB‐231 cells. G) Proximity ligation assay (PLA) analysis of the interaction between TBL2, PRMT5, and WDR77. Scale bar: 10 µm. H) Western blotting analysis of H4R3 dimethylation and SDMA in the indicated cell lines. GAPDH served as a loading control. I) Phosphorylation levels of AKT in vector and TBL2 overexpressed MDA‐MB‐231 cells treated with PBS or GSK3326595 (0.3 or 0.6 µM for 4 days). J) Immunoprecipitation analysis for AKT dimethylation in response to TBL2 overexpression and silence in MDA‐MB‐231 cells. The data shown are representative of three independent experiments.

To investigate potential oncogenic signals regulated by TBL2, a scaffold protein containing a WDR domain, we conducted gene set enrichment analysis (GSEA) on TCGA BC samples based on TBL2 expression levels. Our findings revealed a significant enrichment of genes in the PI3K‐AKT‐mTOR pathway in the high TBL2 expression group, indicating a role for TBL2 in regulating AKT activity (**Figure** **5**A). Subsequent western blotting experiments on stable cell lines overexpressing or silencing TBL2 demonstrated that elevated TBL2 expression upregulated AKT phosphorylation, whereas reduced TBL2 expression had the opposite effect, decreasing both AKT and GSK3β phosphorylation. Notably, alterations in TBL2 expression did not impact the overall expression levels of AKT1, GSK3β, and PDK1 (Figure 5B). Moreover, we observed that insulin‐induced AKT activation was significantly impaired in TBL2‐silenced MCF7 and MDA‐MB‐231 cells (Figure 5C). Together, these findings indicate that TBL2 promotes the phosphorylation of AKT.

### 2.5 TBL2 Interacts with PRMT5 and WDR77

To investigate the role of TBL2 in promoting AKT phosphorylation, we conducted immunoprecipitation‐mass spectrometry (IP‐MS) to identify TBL2‐interacting proteins. 293T and MDA‐MB‐231 cells overexpressing C‐terminal Flag‐tagged TBL2 or luciferase (Luc) were subjected to tandem immunoprecipitation (Figure S2A, Supporting Information). Following the elimination of non‐specific binding proteins from each control group, 522 proteins were found to interact with TBL2 in the 293T‐TBL2‐Flag group, while 308 proteins interacted with TBL2 in the MDA‐MB‐231 group. The intersection between the two groups identified 225 high‐confidence TBL2‐interacting proteins (Table S3, Supporting Information), including PRMT5 and WDR77 (Figure S2B, Supporting Information). Previous research has suggested that PRMT5, a type II methyltransferase, can activate AKT by catalyzing dimethylation of arginine 391 (R391) on AKT1.^[^ ^16^ ^]^ Additionally, PRMT5 and WDR77 have been shown to function together as complexes.^[^ ^23^ ^]^ Given these findings, we hypothesized that TBL2 could affect AKT phosphorylation by modulating PRMT5's methyltransferase activity through its interaction with PRMT5 and WDR77. This hypothesis was tested through endogenous and exogenous immunoprecipitation experiments on MDA‐MB‐231 and 293T cells, respectively, confirming the binding of TBL2 with PRMT5 and WDR77 (Figures 5D,E). Immunofluorescence staining and proximity ligation assay further supported the co‐localization and endogenous protein‐protein interaction of TBL2 with PRMT5 and WDR77 (Figures 5F,G; Figure S2C, Supporting Information).

Furthermore, we validated whether TBL2 affects the methyltransferase activity of PRMT5. H4R3 arginine is a primary substrate of PRMT5, and its methylation level along with SDMA can serve as markers for evaluating PRMT5 methyltransferase activity.^[^ ^23^ ^]^ Western blotting demonstrated that TBL2 overexpression increased H4R3me2s and SDMA levels, while TBL2 knockdown had the opposite effect (Figure 5H). Notably, PRMT5 mRNA and protein expression remained unaltered, indicating that TBL2 may impact PRMT5's enzymatic activity (Figure 5H; Figure S2D, Supporting Information). Additionally, PRMT5 inhibitors GSK3326595 and GSK591 reversed the increase in AKT phosphorylation induced by TBL2 overexpression, suggesting that TBL2 regulates AKT phosphorylation by influencing PRMT5 methyltransferase activity (Figure 5I; Figure S2E, Supporting Information). Furthermore, knocking down WDR77 also reversed the increase in p‐AKT induced by TBL2 overexpression, indicating that TBL2's promotion of AKT phosphorylation is dependent on WDR77 (Figure S2F, Supporting Information). To understand whether TBL2 influences the symmetric dimethylation of AKT in cells, we immunoprecipitated AKT from the lysates of MDA‐MB‐231 cells overexpressing or silencing TBL2. Western blot results showed a corresponding increase in levels of dimethylation and phosphorylation of AKT, whereas reduced TBL2 expression had the opposite effect (Figure 5J). Overall, these findings suggest that TBL2 promotes AKT phosphorylation by modulating the methyltransferase activity of PRMT5.

### 2.6 TBL2 Acts as a Scaffold to Promote the Interaction Between PRMT5 and WDR77

**Figure 6:**
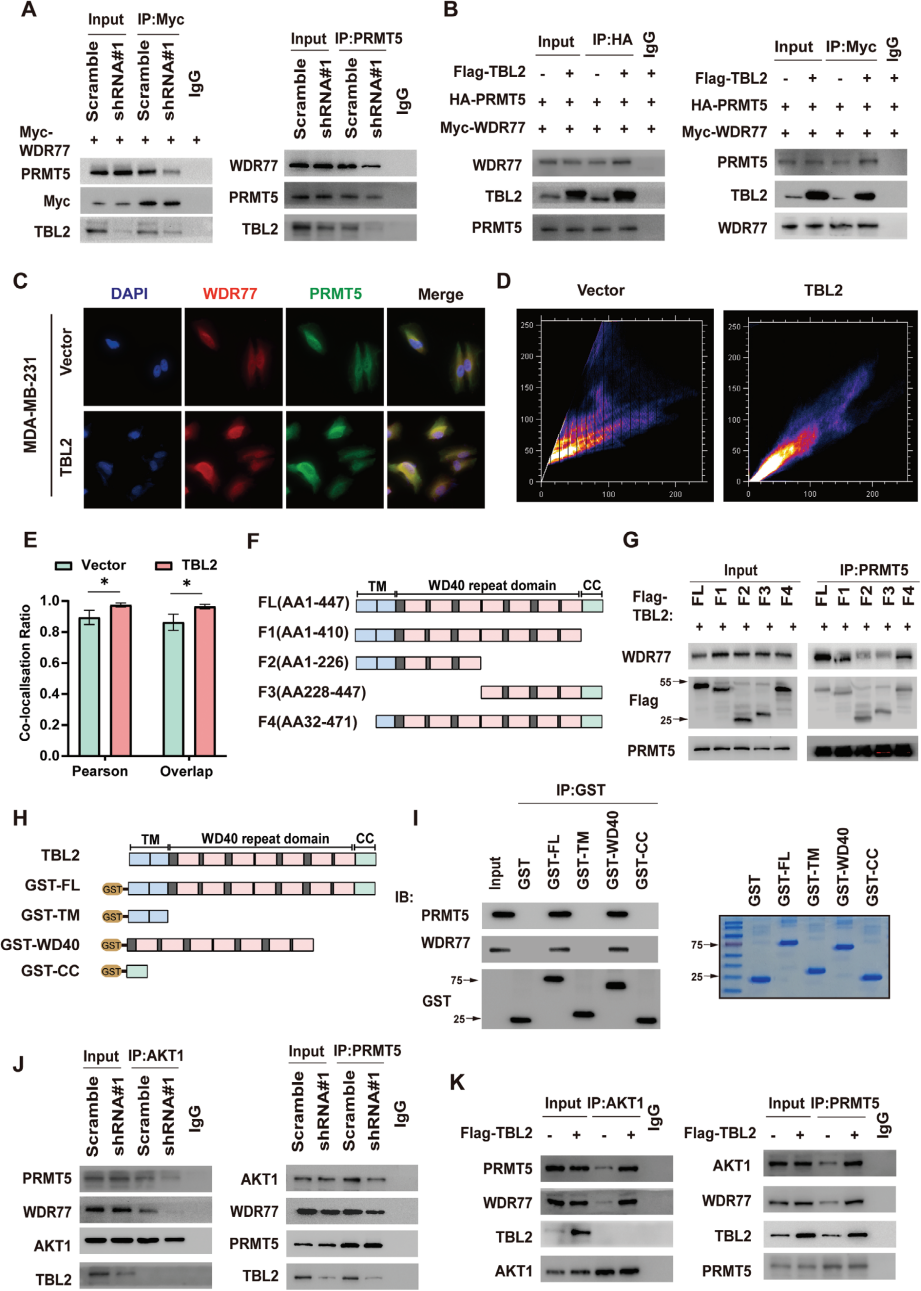
TBL2 Acts as a Scaffold to Promote the Interaction Between PRMT5 and WDR77. A) Reciprocal IP assays of PRMT5 and WDR77 in TBL2‐silenced or non‐silenced MDA‐MB‐231 cells. B) Reciprocal IP assays of PRMT5 and WDR77 in 293T cells transfected or untransfected with Flag‐TBL2. C) Fluorescence immunostaining of PRMT5 and WDR77 in vector or TBL2‐overexpressed MDA‐MB‐231 cells. D) A scatterplot of red and green pixel intensities in two cell types in Figure 6C. E) The Pearson's correlation and overlap coefficient between PRMT5 and WDR77 computed based on data from three independent experiments using the conditions specified in Figure 6C. An estimated 50 cells were examined per experiment. F) Schematic illustration of TBL2 truncated constructs. G) TBL2‐silenced 293T cells were transfected with indicated Flag‐TBL2 truncations, followed by IP assays with PRMT5 to examine the interaction between PRMT5 and WDR77. H) Schematic illustration of GST‐tagged full‐length or deletion mutants of TBL2. I) GST pull‐down assays were carried out to determine the direct interaction between GST‐tagged TBL2 and PRMT5/WDR77. Purified GST and GST‐tagged full‐length or deletion mutants of TBL2 were visualized using Coomassie Brilliant Blue R/G staining solution (right panel). J) Reciprocal IP assays of PRMT5 and AKT1 in TBL2‐silenced or non‐silenced MDA‐MB‐231 cells. K) Reciprocal IP assays of PRMT5 and AKT1 in MDA‐MB‐231 cells transfected or untransfected with Flag‐TBL2. The statistical analysis was performed using a two‐tailed Student's t‐test. The presented data indicate the means ± S.D. obtained from three independent experiments. ^*^ *P* < 0.05.

Previous studies have established that WDR domains typically serve as scaffolds. Therefore, we explored whether TBL2 could function as a scaffolding protein. IP assays were conducted, revealing that TBL2 depletion significantly reduced the endogenous interactions between PRMT5 and WDR77 (**Figure** **6**A). Conversely, TBL2 overexpression substantially enhanced the interactions between these proteins (Figure 6B). The protein grayscale comparison of three independent IP experiments is shown in Figure S3A (Supporting Information), indicating that TBL2 plays a crucial role in promoting their interaction. This point was further corroborated through immunofluorescence staining and fluorescence colocalization‐based quantitative analysis (Figures 6C–E).

To determine whether the WDR domain‐mediated homomeric state is essential for TBL2's scaffolding function, full‐length or truncated constructs of TBL2 were reintroduced into TBL2‐silencing BC cells. The overexpression of the TBL2 truncated body lacking the WD40 repeat domain significantly weakened the interaction, while the binding effect between PRMT5 and WDR77 was significantly enhanced when the full‐length, F1, and F4 truncated bodies were reconstructed (Figures 6F,G). These results indicate that TBL2, as a scaffold protein, promotes the binding of PRTM5 and WDR77 through its WD40 domain. To further validate the interaction and identify the domains responsible, we performed GST pull‐down assays using transgenic HEK293T cells expressing full‐length or truncated GST‐tagged TBL2. Our GST pull‐down analysis demonstrated that the WD40 domain of TBL2 directly interacts with both PRMT5 and WDR77(Figures 6H,I). Additionally, we found that the WD40 domain of TBL2 alone is sufficient to interact with PRMT5 and WDR77 (Figures S3B,C, Supporting Information).

We conducted additional investigations to determine if TBL2 influences the interaction between PRMT5 and AKT1 in BC cells. Our IP experiments revealed a decrease in AKT1 binding to PRMT5 in MDA‐MB‐231 cells following TBL2 knockdown (Figure 6J). Conversely, this binding was enhanced in cells overexpressing TBL2 (Figure 6K). Similarly, WDR77 knockdown prevents the TBL2‐induced increase in PRMT5‐AKT binding, while WDR77 overexpression increases PRMT5‐AKT binding even when TBL2 is knocked down (Figure S3D, Supporting Information). These results suggest that TBL2 mainly mediates the binding of PRMT5 and WDR77, thereby affecting the binding of PRMT5 to the substrate and promoting its methylase ability.

### 2.7 TBL2 Promotion of BC Cell Proliferation is Dependent on PRMT5/WDR77

**Figure 7:**
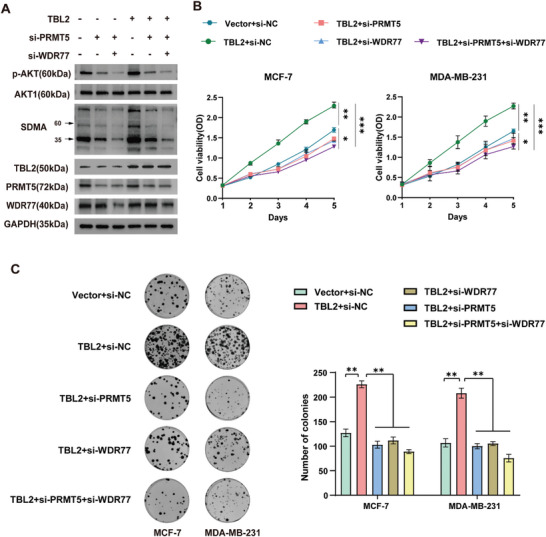
TBL2 promotion of BC cell proliferation is dependent on PRMT5/WDR77. A) Western blot assay was used to analyze the phosphorylation level of AKT in each group of cells. GAPDH served as a loading control. B) CCK8 assay detected the proliferation ability of MCF‐7 and MDA‐MB‐231 conducted in the indicated cells. C) Plate cloning assay was used to detect the proliferation ability of corresponding cells. A two‐tailed Student's t‐test was used, and the data was presented as mean ± S.D. from three independent experiments. ns. no significance, ^*^ *P* < 0.05, ^**^ *P* < 0.01, ^***^ *P* < 0.001.

In addition, western blotting experiments showed that knocking down PRMT5 or WDR77 could completely reverse the increase in AKT phosphorylation induced by TBL2 overexpression in MDA‐MB‐231 cells (**Figure** **7**A). Subsequently, rescue experiments revealed that in the CCK8 experiment, downregulation of PRMT5 or WDR77 compensated for the increased proliferation of MDA‐MB‐231 cells caused by overexpression of TBL2 (Figure 7B). The same results were obtained from the plate cloning experiment (Figure 7C). Moreover, when we exposed TBL2‐deficient MDA‐MB‐231 cells to the AKT‐specific activator SC79, we observed that activated AKT was able to restore the proliferation capacity of these cells, providing further evidence that TBL2 impacts breast cancer cell proliferation via the AKT pathway (Figures S3E–G, Supporting Information). In summary, these results indicate that TBL2 promotion of BC cell proliferation is dependent on PRMT5/WDR77.

## 3 Discussion

Cell proliferation, the process driving cancer cells’ multiplication and tumor formation, is a critical factor in cancer progression, particularly BC.^[^ ^24^, ^25^ ^]^ Uncontrolled cell proliferation fuels the growth of cancerous tissues and metastasis, emphasizing the importance of understanding and targeting proliferation pathways for therapeutic interventions.^[^ ^26^ ^]^ Our study observed an overexpression of TBL2 in BC, correlating with an elevated risk of tumor recurrence and reduced patient survival rates. Importantly, inhibiting TBL2 has shown significant potential in impeding BC cell proliferation, highlighting it as a promising therapeutic target for this disease.

PRMT5, a major type II arginine methyltransferase, catalyzes the dimethylation of histone H3 arginine 8 (H3R8me2s) and H4 arginine 3 (H4R3me2s) and acts as an epigenetic regulatory factor to regulate gene transcription.^[^ ^27^ ^]^ PRMT5 influences genome transcription, cell differentiation, the cell cycle, and spliceosome assembly through symmetric ω‐guanidine di‐methylation of both ends of arginine residues on the substrate proteins.^[^ ^28^ ^]^ PRMT5 overexpression is observed in various cancer types, contributing to the regulation of transcription factors and growth pathways that sustain tumor stem cells.^[^ ^29^, ^30^, ^31^ ^]^ Recent findings indicate that PRMT5 methylation is responsible for AKT phosphorylation at Thr308 and Ser473.^[^ ^16^, ^17^ ^]^ Consistently, our study uncovered that TBL2 overexpression increased PRMT5's methylase activity, leading to higher levels of AKT phosphorylation. Moreover, treatment with the PRMT5‐specific small molecule inhibitors GSK3326595 and GSK591 effectively countered the heightened AKT phosphorylation levels caused by TBL2 overexpression. These results further confirm that PRMT5 promotes AKT phosphorylation and activation through AKT methylation. However, the specific site of PRMT5's action on AKT requires further investigation.

WDR77, also known as methylosome protein 50 (MEP50), serves as the primary protein partner of PRMT5, influencing its catalytic activity and substrate specificity.^[^ ^32^ ^]^ The PRMT5/WDR77 complex exhibits higher methyltransferase activity than PRMT5 alone, potentially due to WDR77's positive allosteric effect on the binding of PRMT5 to proteins or SAM substrates or its role in presenting protein substrates to PRMT5^[^ ^33^ ^]^ Overexpression of WDR77 is associated with poor prognosis in lung and breast cancer,^[^ ^34^, ^35^, ^36^ ^]^ making the PRMT5/WDR77 complex an attractive therapeutic target. Moreover, PRMT5‐specific inhibitors are currently in preclinical and clinical development.^[^ ^37^, ^38^ ^]^ Interestingly, our study found that TBL2 utilizes its WDR domain as a scaffold platform to facilitate the binding of PRMT5 to WDR77. This interaction enhances the methylase activity of PRMT5 and strengthens its binding to AKT1, consequently promoting BC cell proliferation. Notably, Our study found that TBL2 promotes AKT phosphorylation activation and cell proliferation dependent on PRMT5/WDR77, which is consistent with previous findings.^[^ ^39^, ^40^ ^]^ These findings suggest that inhibiting the binding of PRMT5 to WDR77 may emerge as a more effective strategy to curtail PRMT5's methylase activity. While our study provides valuable insights into the regulatory role of TBL2 in promoting the interaction between PRMT5 and WDR77, it is important to acknowledge certain limitations. Specifically, while our protein spectrum analysis and CO‐IP experiments suggest direct interactions between TBL2, PRMT5, and WDR77, we did not identify the specific domains responsible for mediating these interactions. Future studies should aim to address this aspect to gain a deeper understanding of the molecular mechanisms underlying these interactions. By elucidating the precise domains involved, we can further explore the functional implications of TBL2's regulatory role in breast cancer progression. Besides, the response experiments in our study were not validated in vivo.

Protein‐protein interactions (PPIs) are crucial for the control of biological systems.^[^ ^41^, ^42^ ^]^ Recent advancements highlight certain PPIs as potential therapeutic targets, with the development of the first PPI inhibitors reaching clinical stages.^[^ ^43^, ^44^, ^45^ ^]^ The WDR domain, abundant in the human proteome, with its distinctive doughnut structure, easily binds to drug‐like small molecules with high affinity, becoming a new potential target for drug discovery.^[^ ^46^ ^]^ As a member of the β‐transduction protein family of the WDR domain, TBL2 is intricately involved in intracellular signal transduction pathways.^[^ ^47^ ^]^ Previous studies have indicated that TBL2 preferentially interacts with the phosphorylated PERK and is involved in inducing ATF4 expression during ER stress, suggesting that TBL2 may play a protective role under cellular stress conditions.^[^ ^19^, ^48^ ^]^ Our study discovered that TBL2 promoted the binding between PRMT5 and WDR77 by utilizing its WDR domain as a scaffold platform. When TBL2 is either knocked down or its WDR domain disrupted, this interaction is inhibited, resulting in reduced methylase activity of PRMT5. These findings introduce a fresh perspective for drug design based on the structural attributes of the WDR domain, offering promising avenues for therapeutic advancements.

## 4 Conclusions

In summary, our research has identified the role of TBL2 in promoting BC cell proliferation by increasing PRMT5 methyltransferase activity. This, in turn, enhances AKT phosphorylation and activates downstream cascade signaling. The results and conclusion of our study contribute to a better understanding of the dysregulation of BC cell proliferation and present potential targets for innovative interventions against this disease.

## 5 Experimental Section

### Patient Information and Tissue Specimens

Clinical data from the Sun Yat‐Sen University Cancer Center's electronic medical records from 2005 to 2017 were examined. The study included 200 patients with histopathologically confirmed invasive ductal carcinoma, for whom surveillance data were available. Additionally, 10 tumor‐adjacent samples were included for comparative analysis. A summary of clinicopathologic features is presented in Table S1 (Supporting Information). The study was ethically authorized by the Sun Yat‐Sen University Cancer Center's ethical committee.

### Quantitative Real‐Time PCR

RNA extraction was performed using the TRIzol kit (Thermo Fisher Scientific). Reverse transcription was used to generate cDNA from 2 micrograms of RNA with RNase‐free DNase. Quantitative PCR (qPCR) was carried out using the CFX96 Real‐Time PCR Detection System, SYBR premix Ex Taq (Takara), and one microliter of cDNA (Bio‐Rad). Relative expression levels were determined by contrasting the Ct value of target genes with GAPDH.

### Western Blotting

Cellular proteins were extracted using a lysis buffer for the radioimmunoprecipitation test (Thermo Fisher Scientific). Protein concentration was assessed using a Bio‐Rad DC protein assay kit II. Proteins were separated on 7.5% to 15% SDS‐PAGE gels, transferred onto polyvinylidene fluoride (PVDF) transfer membranes, and blocked with 5% BSA. The membrane was incubated overnight with primary antibodies at 4 °C, followed by tagged secondary antibodies. Immunoreactive proteins were visualized using an ECL chemiluminescent substrate reagent kit (Thermo Fisher Scientific). Antibodies used included anti‐TBL2 (12488‐1‐AP, Proteintech, 1:1000), anti‐PRMT5 (18436‐1‐AP, Proteintech, 1:1000), anti‐WDR77 (200955, Zen BioScience, 1:1000), anti‐p‐AKT (Ser473) (#4060, CST, 1:1000), anti‐AKT1 (#2938, CST, 1:1000), anti‐p‐AKT (Thr308) (#13038, CST, 1:1000), anti‐H4R3me2s (A3159, Abclonal, 1:1000), anti‐SDMA (#13222, CST, 1:1000), anti‐p‐GSK‐3β (Ser9) (#9323, CST, 1:1000) and anti‐GAPDH (#5174, CST, 1:2000).

### Immunohistochemistry

Immunohistochemical (IHC) staining was conducted on 200 breast cancer specimens. Briefly, paraffin‐embedded specimens were cut into 4 µm sections, baked at 65 °C for 30 min, deparaffinized, and rehydrated with xylene. The sections were immersed in EDTA antigen extraction buffer and heated for antigen repair. Samples were then incubated with primary antibodies for overnight at 4 °C. Following washing, the tissue slices were treated with a biotinylated secondary antibody (Zsbio, BJ, China). The slices were then dehydrated, mounted in Crystal Mount, counterstained with 10% Mayer's hematoxylin, and submerged in 3‐amino‐9‐ethyl carbazole. Staining was scored independently by two blinded pathologists who were unaware of the clinical outcome. TBL2 staining received four scores: strong + 3, middling + 2, weak + 1, and negative 0. High expression was defined as samples with scores of + 3 or + 2; low expression was defined as samples with + 1 or 0 scores. Antibodies used included anti‐TBL2 (12488‐1‐AP, Proteintech, 1:100) and anti‐KI67 (A20018, Abclonal, 1:200).

### Creating Cell Lines with Stable Transfection

Lipofectamine 3000 (Thermo Fisher Scientific) was used for transient plasmid transfection. Human breast cancer cell lines (MCF‐7, MDA‐MB‐231) and the mouse‐derived BC cell line (4T1) were infected with a retrovirus plasmid encoding two distinct short hairpin RNAs targeting TBL2 to create stable TBL2‐knockdown cells (Kidan, Guangzhou, China). shRNA sequences were as follows: shRNA#1, 5′‐GGTGCGAGCCTTCGAACTAAA‐3′; shRNA#2, 5′‐ TTGGTGTTGATGGTAGACAGC‐3′. The pcdh‐cmv‐mcs‐ef1‐puro vector contained TBL2 and its shortened variants, either with or without affinity‐tagged bait proteins (Clontech, Mountain View, CA, USA) for overexpression. The lentiviruses containing TBL2 were used for overexpression in MCF‐7, MDA‐MB‐231, and 4T1 cells. Puromycin was used to identify the positive clones.

### Xenograft Tumor Models

Six animals per group were randomly assigned to 4 groups. Animals were injected stably transfected MDA‐MB‐231 cells (1 × 10^6^ cells/50 µL) or 4T1 cells (2 × 10^5^ cells/50 µL) into the mammary adipose pad. Tumor volumes were measured, and BLI using the Xenogen IVIS Spectrum Imaging System tracked tumor load sporadically every week until the end of the experiment. The data were presented as the mean ± S.D. The Institutional Animal Care and Use Committee at Sun Yat‐Sen University approved the animal studies (Approval No. L102022022030J).

### Co‐Immunoprecipitation Assay

Cancer cells were lysed in lysis buffer (150 mm NaCl, 10 mm HEPES, pH 7.4, 1% NP‐40) containing a combination of protease inhibitors. Anti‐PRMT5 antibodies (18436‐1‐AP, Proteintech) or Anti‐AKT1(#2938, CST, 1:50) were incubated with protein G agarose, Flag, or HA affinity agarose (Sigma–Aldrich) overnight at 4 °C. Immunocomplexes were washed five times with IP wash buffer (150 mm NaCl, 10 mm HEPES, pH 7.4, 0.1% NP‐40) and eluted with 1 m glycine (pH 3.0). The eluates were then combined with sample buffer, denatured, and analyzed by western blotting.

### Immunofluorescence Staining (IF)

Coverslips were seeded with 5 × 10^4^ cells per well. After 24 h, cells were washed thrice with PBS and fixed with 4% (v/v) paraformaldehyde for 15 min. Subsequently, the cells were permeabilized and blocked using a solution containing 0.2% (v/v) Triton X‐100 and 1% (w/v) BSA in PBS for 1 h. Cells were then stained with the following primary antibodies: anti‐TBL2 (1:100, mouse monoclonal, Proteintech, or 1:100, rabbit polyclonal, Proteintech), anti‐PRMT5 (1:100, rabbit polyclonal, Proteintech) and anti‐WDR77 (1:100, mouse polyclonal, Zen BioScience) for 2 h at 4 °C. Secondary antibodies used were goat anti‐mouse Alexa Fluor 488 or goat anti‐rabbit Alexa Fluor 594 (1:1000, CST), and incubated for 1h. Following three PBS washes, cells were counterstained with DAPI (Sigma–Aldrich) to enable visualization of the nuclei. Thresholds were uniformly applied across all conditions to filter noise. Once the thresholds were set, the co‐localization tool in Image J was employed to determine the percentage of co‐localization and Pearson's correlation between respective channels, with at least 50 cells analyzed for each co‐localization study.

### Proximity Ligation Assay

The proximity ligation experiment was conducted following the manufacturer's instructions using a Rabbit PLUS and Mouse MINUS Duolinkinsitu PLA kit (Sigma–Aldrich). In short, the chosen cells on coverslips were fixed for 15 min at room temperature with 3.7% formaldehyde in PBS. After being cleaned with TBS, they were left for 2 h at room temperature in a humidified environment, blocked in 1% BSA in TBST. The coverslips containing cells were treated with anti‐TBL2 (1:100, mouse monoclonal, Proteintech, or 1:100, rabbit polyclonal, Proteintech), anti‐PRMT5 (1:100, rabbit polyclonal, Proteintech) and anti‐WDR77 (1:100, mouse polyclonal, Zen BioScience) antibodies for a whole night at 4 °C. After TBST washing, proximity ligation was performed using the PLAkit (Sigma–Aldrich). The cells were subsequently counterstained with DAPI (Sigma–Aldrich) to reveal the nuclei. PLA signals were detected using an Olympus BX51 microscope (Olympus, Tokyo, Japan) under ×40 objectives.

### Statistical Analysis

SPSS version 19.0 was utilized for conducting statistical analysis. The log‐rank test, χ2 test, Spearman‐rank correlation test, and two‐tailed Student's t‐test were among the statistical tests that were employed for data analysis. Cox regression analysis was used for multivariate statistical analysis. Statistical significance was set at *P* < 0.05, denoted as follows: ^*^, *P* < 0.05; ^**^, *P* < 0.01; ^***^, *P* < 0.001.

Additional information is provided in the Supplementary Materials and Methods.

### Ethics Approval and Consent to Participate

Ethics approval and prior patient consent had been obtained from the Institutional Research Ethics Committee for the use of the clinical specimens for research purposes.

### Availability of Data and Material

The TCGA data used in this study is publicly available and can be accessed through the official TCGA Data Portal (https://portal.gdc.cancer.gov/) or other authorized databases. The GEO database is publicly available using the provided accession number GSE42568 and GSE103512.

## Conflict of Interest

The authors declare no conflict of interest.

## Author Contributions

X.L., C.Z., L.Z., and S.W. contributed equally to this work. X.L., C.Z., L.Z., and S.W. conceived the idea, designed the experiments, analyzed, and interpreted the data, and was a major contributor in writing the manuscript. X.L., Y.L., C.Z., and S.W. designed and performed the experiments, generated the data, and contributed to the manuscript preparation. W.Z. and X.W. performed the immunostaining of the tissue. L.Z. and Q.Y. performed the bioinformatics analysis. H.T. and S.C. validation and supervision of the manuscript. W.W., T.Y., and L.Y. funded the work and contributed to the manuscript preparation. All the authors read and approved the manuscript.

## Supporting information



Supporting Information

## Data Availability

The data that support the findings of this study are openly available in The Cancer Genome Atlas Program at https://www.cancer.gov/ccg/research/genome-sequencing/tcga, reference number 1. These data were derived from the following resources available in the public domain: [Resource 1], https://www.cancer.gov/ccg/research/genome-sequencing/tcga; [Resource 2], https://www.ncbi.nlm.nih.gov/geo/.

## References

[1] H. Sung , J. Ferlay , R. L. Siegel , M. Laversanne , I. Soerjomataram , A. Jemal , F. Bray , Ca‐Cancer J. Clin. 2021, 71, 209. 33538338 10.3322/caac.21660

[2] N. Harbeck , M. Gnant , Lancet 2017, 389, 1134. 27865536 10.1016/S0140-6736(16)31891-8

[3] S. K. Yeo , J. Guan , Trends Cancer 2017, 3, 753. 29120751 10.1016/j.trecan.2017.09.001 PMC5802368

[4] A. J. Kerr , D. Dodwell , P. Mcgale , F. Holt , F. Duane , G. Mannu , S. C. Darby , C. W. Taylor , Cancer Treat. Rev. 2022, 105, 102375. 35367784 10.1016/j.ctrv.2022.102375 PMC9096622

[5] F. Ye , S. Dewanjee , Y. Li , N. K. Jha , Z. S. Chen , A. Kumar , Vishakha, T. B. , S. K. Jha , H. Tang , Mol. Cancer 2023, 22, 105. 37415164 10.1186/s12943-023-01805-y PMC10324146

[6] Y. Zeng , W. Du , Z. Huang , S. Wu , X. Ou , J. Zhang , C. Peng , X. Sun , H. Tang , Cell Death Discov. 2023, 9, 153. 37160894 10.1038/s41420-023-01448-4 PMC10169853

[7] S. Wu , J. Lu , H. Zhu , F. Wu , Y. Mo , L. Xie , C. Song , L. Liu , X. Xie , Y. Li , H. Lin , H. Tang , Cancer Lett. 2024, 581, 216508. 38029538 10.1016/j.canlet.2023.216508

[8] S. Revathidevi , A. K. Munirajan , Semin. Cancer Biol. 2019, 59, 80. 31173856 10.1016/j.semcancer.2019.06.002

[9] Z. Cai , J. Wang , Y. Li , Q. Shi , L. Jin , S. Li , M. Zhu , Q. Wang , L. L. Wong , W. Yang , H. Lai , C. Gong , Y. Yao , Y. Liu , J. Zhang , H. Yao , Q. Liu , Sci. China Life Sci. 2023, 66, 94. 35982377 10.1007/s11427-021-2140-8

[10] C. Chan , U. Jo , A. Kohrman , A. H. Rezaeian , P. Chou , C. Logothetis , H. Lin , Cell Biosci. 2014, 4, 59. 25309720 10.1186/2045-3701-4-59 PMC4192732

[11] J. He , F. Zeng , X. I. Jin , L. Liang , M. Gao , W. Li , G. Li , Y. Zhou , Oncol. Res. 2023, 31, 615. 37415737 10.32604/or.2023.029698 PMC10319584

[12] A. M. Scherbakov , A. A. Basharina , D. V. Sorokin , E. I. Mikhaevich , I. E. Mizaeva , A. L. Mikhaylova , T. A. Bogush , M. A. Krasil'Nikov , Cancer Drug Resist. 2023, 6, 103. 37065867 10.20517/cdr.2022.96 PMC10099602

[13] B. D. Manning , A. Toker , Cell 2017, 169, 381. 28431241 10.1016/j.cell.2017.04.001 PMC5546324

[14] G. Hoxhaj , B. D. Manning , Nat. Rev. Cancer 2020, 20, 74. 31686003 10.1038/s41568-019-0216-7 PMC7314312

[15] Y. He , M. M. Sun , G. G. Zhang , J. Yang , K. S. Chen , W. W. Xu , B. Li , Signal Transd. Targeted Ther. 2021, 6, 425. 10.1038/s41392-021-00828-5 PMC8677728 34916492

[16] S. Yin , L. Liu , C. Brobbey , V. Palanisamy , L. E. Ball , S. K. Olsen , M. C. Ostrowski , W. Gan , Nat. Commun. 2021, 12, 3444. 34103528 10.1038/s41467-021-23833-2 PMC8187744

[17] L. Huang , X. Zhang , E. J. Rozen , X. Sun , B. Sallis , O. Verdejo‐Torres , K. Wigglesworth , D. Moon , T. Huang , J. P. Cavaretta , G. Wang , L. Zhang , J. M. Shohet , M. M. Lee , Q. Wu , Nat. Commun. 2022, 13, 3955. 35803962 10.1038/s41467-022-31645-1 PMC9270419

[18] C. Behrends , M. E. Sowa , S. P. Gygi , J. W. Harper , Nature 2010, 466, 68. 20562859 10.1038/nature09204 PMC2901998

[19] Y. Tsukumo , S. Tsukahara , A. Furuno , S. Iemura , T. Natsume , A. Tomida , PLoS One 2014, 9, e112761. 25393282 10.1371/journal.pone.0112761 PMC4231078

[20] W. S. Mak , T. M. Tsang , T. Y. Chan , G. L. Lukov , Proteomes 2021, 9, 40. 34698247 10.3390/proteomes9040040 PMC8544692

[21] K. Kosai , T. Masuda , A. Kitagawa , T. Tobo , Y. Ono , Y. Ando , J. Takahashi , N. Haratake , M. Kohno , T. Takenaka , T. Yoshizumi , K. Mimori , Ann. Surg. Oncol. 2023, 30, 7538. 37477745 10.1245/s10434-023-13864-y

[22] Y. Li , S. Liu , Y. Wang , H. Min , D. Adi , X. Li , Y. Yang , Z. Y. Fu , Y. Ma , Lipids Health Dis. 2020, 19, 186. 32811528 10.1186/s12944-020-01359-8 PMC7433086

[23] S. Antonysamy , Sub‐Cell. Biochem. 2017, 83, 185. 10.1007/978-3-319-46503-6_7 28271477

[24] D. Hanahan , Cancer Discov. 2022, 12, 31. 35022204 10.1158/2159-8290.CD-21-1059

[25] Y. Man , C. Mannion , A. Stojadinovic , G. E. Peoples , W. C. Cho , S. W. Fu , X. Tan , Y. Hsiao , A. Liu , A. Semczuk , P. Zarogoulidis , A. B. Gapeev , X. Deng , X. Peng , B. A. Reva , T. Omelchenko , J. Wang , G. Song , T. Chen , J. Cancer 2023, 14, 573. 37057291 10.7150/jca.82291 PMC10088539

[26] Y. Zou , F. Ye , Y. Kong , X. Hu , X. Deng , J. Xie , C. Song , X. Ou , S. Wu , L. Wu , Y. Xie , W. Tian , Y. Tang , C. W. Wong , Z. S. Chen , X. Xie , H. Tang , Adv. Sci. 2023, 10, e2203699. 10.1002/advs.202203699 PMC9929130 36529697

[27] H. Shailesh , Z. Z. Zakaria , R. Baiocchi , S. Sif , Oncotarget 2018, 9, 36705. 30613353 10.18632/oncotarget.26404 PMC6291173

[28] N. Stopa , J. E. Krebs , D. Shechter , Cell. Mol. Life Sci. 2015, 72, 2041. 25662273 10.1007/s00018-015-1847-9 PMC4430368

[29] X. Wang , T. Qiu , Y. Wu , C. Yang , Y. Li , G. Du , Y. He , W. Liu , R. Liu , C. Chen , Y. Shi , J. Pan , J. Zhou , D. Jiang , C. Chen , Cell Death Differ. 2021, 28, 2931. 33972717 10.1038/s41418-021-00793-0 PMC8481478

[30] E. Beketova , J. L. Owens , A. M. Asberry , C. Hu , Cancer Gene Ther. 2022, 29, 264. 33854218 10.1038/s41417-021-00327-3 PMC8514585

[31] J. Zheng , B. Li , Y. Wu , X. Wu , Y. Wang , J. Med. Chem. 2023, 66, 8407. 37366223 10.1021/acs.jmedchem.3c00250

[32] E. S. Burgos , C. Wilczek , T. Onikubo , J. B. Bonanno , J. Jansong , U. Reimer , D. Shechter , J. Biol. Chem. 2015, 290, 9674. 25713080 10.1074/jbc.M115.636894 PMC4392268

[33] A. M. Asberry , X. Cai , X. Deng , U. Santiago , S. Liu , H. S. Sims , W. Liang , X. Xu , J. Wan , W. Jiang , C. J. Camacho , M. Dai , C. Hu , J. Med. Chem. 2022, 65, 13793. 36206451 10.1021/acs.jmedchem.2c01000 PMC11167723

[34] Y. Peng , Y. Li , L. L. Gellert , X. Zou , J. Wang , B. Singh , R. Xu , L. Chiriboga , G. Daniels , R. Pan , D. Y. Zhang , M. J. Garabedian , R. J. Schneider , Z. Wang , P. Lee , J. Cell. Mol. Med. 2010, 14, 2780. 19840198 10.1111/j.1582-4934.2009.00936.x PMC3822728

[35] R. Liu , J. Gao , Y. Yang , R. Qiu , Y. Zheng , W. Huang , Y. Zeng , Y. Hou , S. Wang , S. Leng , D. Feng , W. Yu , G. Sun , H. Shi , X. Teng , Y. Wang , Nucleic Acids Res. 2018, 46, 6608. 29846670 10.1093/nar/gky461 PMC6061854

[36] S. Suresh , M. Vinet , R. Dakroub , L. Lesage , M. Ye , H. Fayyad‐Kazan , A. Nicolas , D. Meseure , T. Dubois , Cancers 2022, 14, 4766. 36230689 10.3390/cancers14194766 PMC9563057

[37] Q. Wu , M. Schapira , C. H. Arrowsmith , D. Barsyte‐Lovejoy , Nat. Rev. Drug Discov. 2021, 20, 509. 33742187 10.1038/s41573-021-00159-8

[38] S. Fu , Q. Zheng , D. Zhang , C. Lin , L. Ouyang , J. Zhang , L. Chen , Eur. J. Med. Chem. 2022, 244, 114842. 36274274 10.1016/j.ejmech.2022.114842

[39] Z. Gu , F. Zhang , Z. Wang , W. Ma , R. E. Davis , Z. Wang , Oncogene 2013, 32, 1888. 22665061 10.1038/onc.2012.207 PMC3935615

[40] H. Qi , X. Shi , M. Yu , B. Liu , M. Liu , S. Song , S. Chen , J. Zou , W. Zhu , J. Luo , J. Biol. Chem. 2018, 293, 17769. 30282801 10.1074/jbc.RA118.003629 PMC6240876

[41] A. Athanasios , V. Charalampos , T. Vasileios , G. Ashraf , Curr. Drug Metab. 2017, 18, 5. 28889796 10.2174/138920021801170119204832

[42] M. Mou , Z. Pan , Z. Zhou , L. Zheng , H. Zhang , S. Shi , F. Li , X. Sun , F. Zhu , Research 2023, 6, 240. 10.34133/research.0240 PMC10528219 37771850

[43] D. A. Abed , M. Goldstein , H. Albanyan , H. Jin , L. Hu , Acta Pharm. Sin. B 2015, 5, 285. 26579458 10.1016/j.apsb.2015.05.008 PMC4629420

[44] D. E. Scott , A. R. Bayly , C. Abell , J. Skidmore , Nat. Rev. Drug Discov. 2016, 15, 533. 27050677 10.1038/nrd.2016.29

[45] C. Liu , Y. Zhang , J. Gao , Q. Zhang , L. Sun , Q. Ma , X. Qiao , X. Li , J. Liu , J. Bu , Z. Zhang , L. Han , D. Zhao , Y. Yang , Drug Resist. Updat. 2023, 66, 100903. 36463808 10.1016/j.drup.2022.100903

[46] M. Schapira , M. Tyers , M. Torrent , C. H. Arrowsmith , Nat. Rev. Drug Discov. 2017, 16, 773. 29026209 10.1038/nrd.2017.179 PMC5975957

[47] L. A. Pérez Jurado , Y. K. Wang , U. Francke , J. Cruces , Cytogenet. Genome Res. 1999, 86, 277. 10.1159/000015319 10575226

[48] Y. Tsukumo , S. Tsukahara , A. Furuno , S. Iemura , T. Natsume , A. Tomida , J. Cell. Biochem. 2016, 117, 500. 26239904 10.1002/jcb.25301

