# Supplementary material for: TBL2 Promotes Tumorigenesis via PRMT5/WDR77‐Mediated AKT Activation in Breast Cancer
Source: Adv Sci (Weinh). 2024 Nov 5;11(47):2400160. doi: 10.1002/advs.202400160 (PMC11653647; doi:10.1002/advs.202400160)
Supplement: Supplementary file 1 — Supporting Information [file ADVS-11-2400160-s001.docx]

**Supplementary information**

**Supplementary Figures**

**
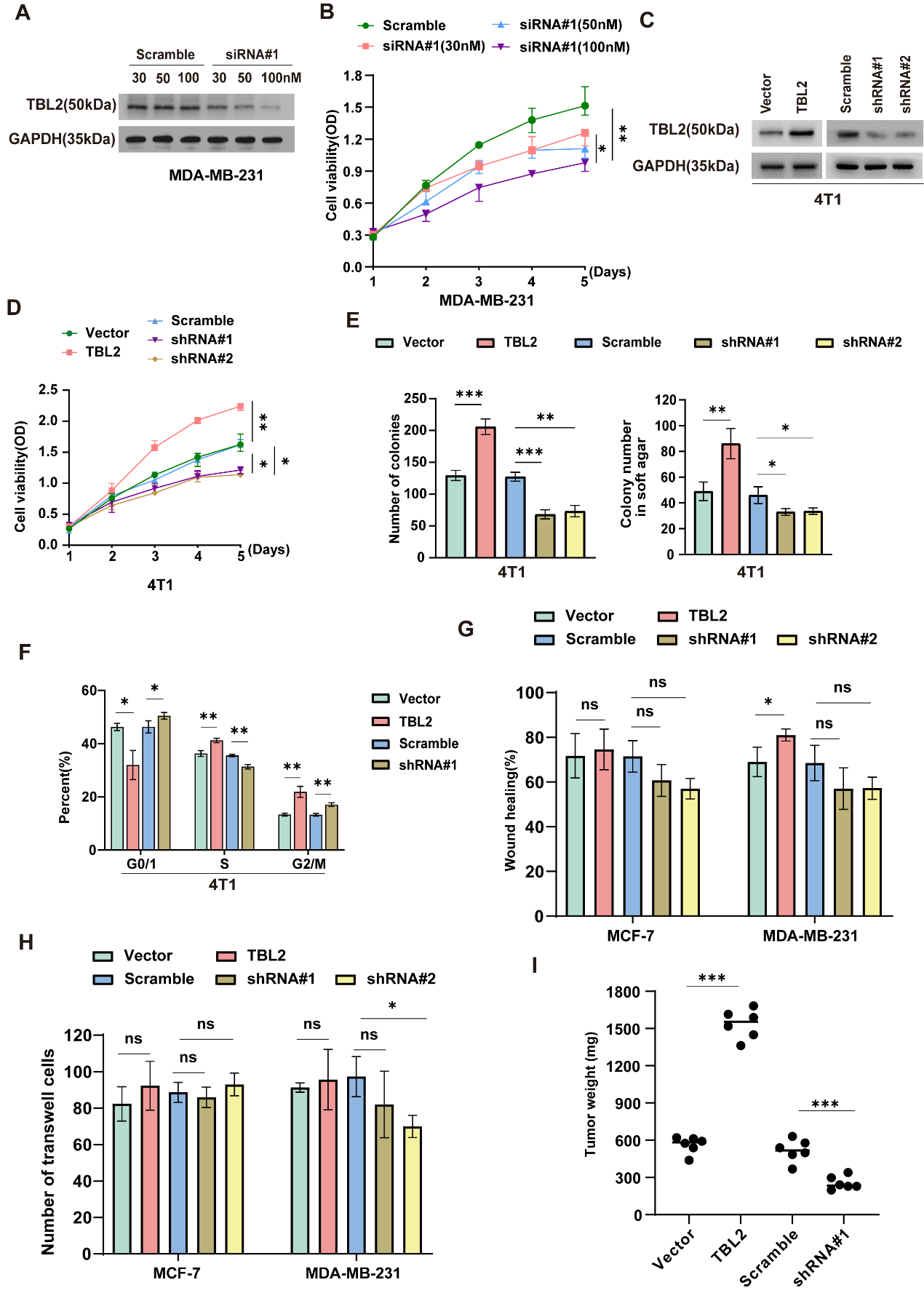
**

**Figure S1** (A)Western blotting analysis of TBL2 in MDA-MB-231 transfected increasing dose of TBL2siRNA (0nM, 30nM, 50nM, and 100nM). (B) CCK-8 assay of MDA-MB-231 transfected with increasing dose of TBL2siRNA (0nM, 30nM, 50nM, and 100nM). (C) Western blotting analysis of TBL2 in 4T1 cells stably transduced with TBL2-overexpressing and TBL2-silencing plasmids. GAPDH was utilized as a control for protein loading. (D-H) CCK-8 (D), colony formation (E), flow cytometric analysis (F), wound healing (G) and transwell (H) were performed in the indicated cells. A two-tailed Student’s t-test was used. (I) Comparison of tumor weight in four groups (n = 6). The presented data indicate the means ± S.D. obtained from three independent experiments, **P* < 0.05; ***P* < 0.01; ****P* <0.001.

**
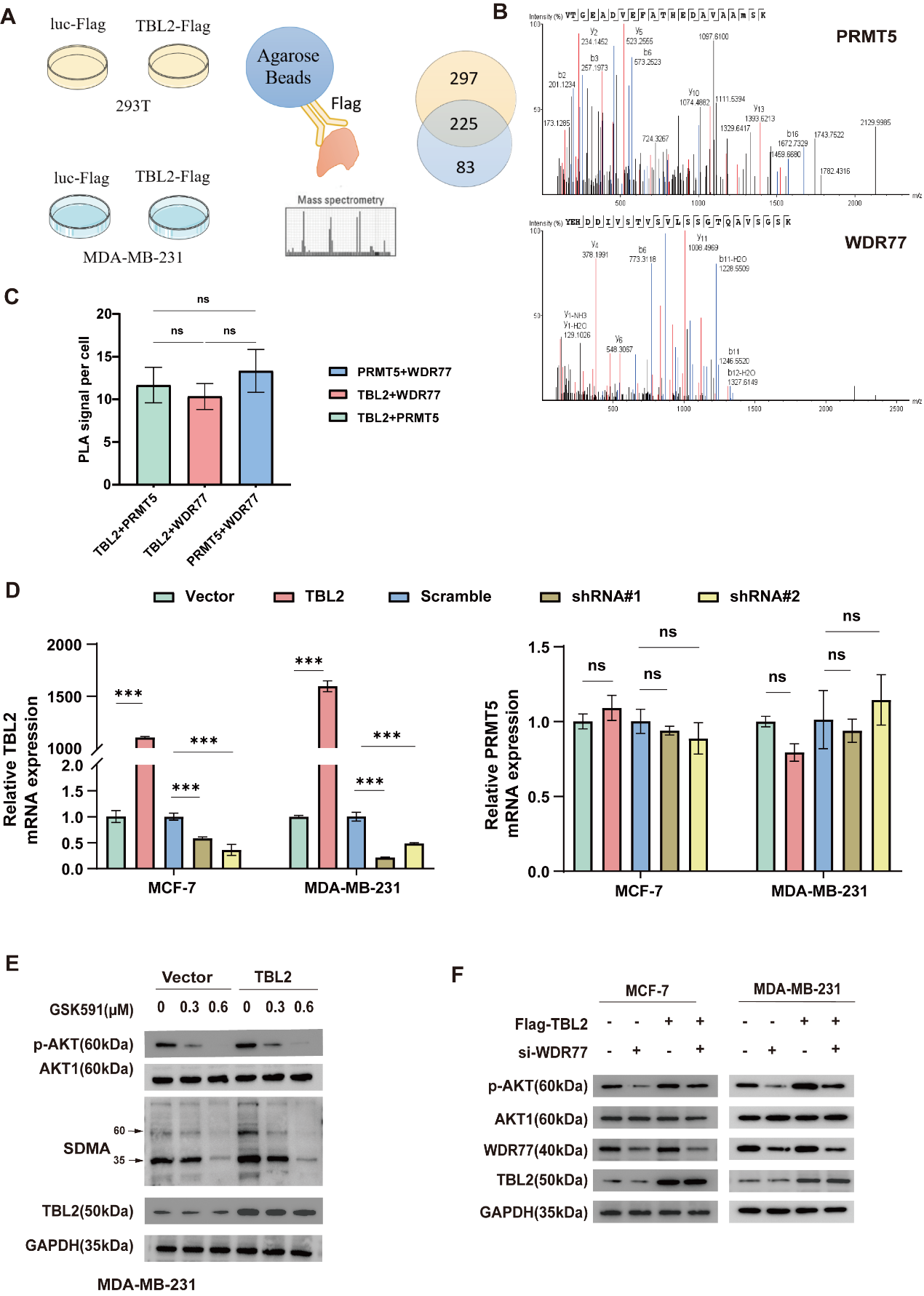
**

**Figure S2** (A) Experimental model of interaction protein mass spectrometry. (B) Secondary mass spectrum of PRMT5 and WDR77. (C) The PLA signal was quantified by counting the foci in 5 random fields per cell. Data were plotted as the mean ± SD of biological triplicates. (D) qRT-PCR analyses of TBL2(left panel) and PRMT5(right panel) expression in the indicated cells. (E) Phosphorylation levels of AKT in vector and TBL2 overexpressed MDA-MB-231 cells treated with PBS or GSK591 (2.5μM or 5.0μM for 4days). (F) Western blotting analysis of TBL2, p-AKT, AKT1, WDR77, TBL2 in TBL2-transduced cells and si-WDR77 cells. GAPDH was used as a control for protein loading. The presented data indicate the means ± S.D. obtained from three independent experiments, ns, no significance; **P* < 0.05; ***P* < 0.01; ****P* <0.001.


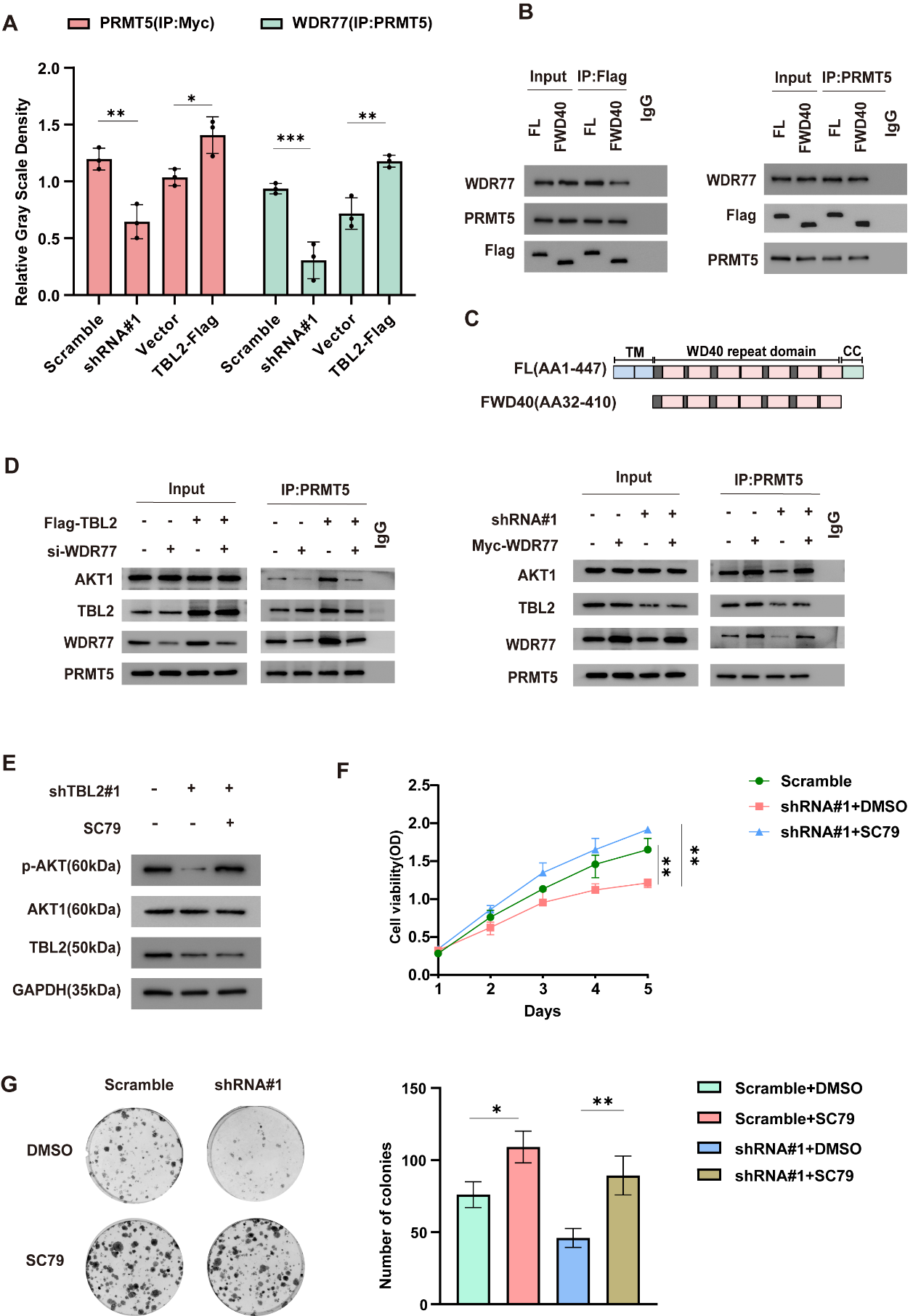


**Figure S3** (A) Relative gray scale density was measured (ImageJ software), and normalized to Myc or PRMT5. A two-tailed Student’s t-test was used. (B) TBL2-silenced 293T cells were transfected with indicated Flag-TBL2 truncations, followed by IP assays with Flag to examine the interaction between TBL2, PRMT5 and WDR77. (C) Schematic illustration of TBL2 truncated constructs. (D) IP assays of PRMT5 and AKT1 in MDA-MB-231 cells overexpressed or knock-downed TBL2 or WDR77. (E) MDA-MB-231 cells were treated with SC79 (10 μmol/L) or DMSO for 48h in the control and TBL2 knockdown groups, and the levels of p-AKT, AKT1, TBL2 were detected using Western blotting analysis. GAPDH was utilized as a control for protein loading. (F-G) CCK8 assay(F) and colony formation(G) detected the proliferation ability of MDA-MB-231 cells were treated with SC79 (10 μmol/L) or DMSO for 48h in the control and TBL2 knockdown groups. A two-tailed Student’s t-test was used, and the data was presented as mean ± S.D. from three independent experiments. ns. no significance, **P* < 0.05; ***P* < 0.01; ****P* <0.001.

**Supplementary Materials and Methods**

**Cells**

Breast cancer cell lines were procured from ATCC. MCF-7, HCC1599, SK-BR-3, and MDA-MB-231 cells were propagated in DMEM medium supplemented with 10% FBS. BT-549, ZR-75-1 and 4T1 cells were maintained in RPMI-1640 medium containing 10% FBS. Ham's F-12 medium supplemented with 10% FBS was used to maintain SUM159PT cells. All the cell lines were enhanced with hydro-cortisone, insulin, penicillin/streptomycin, HEPES, and L-glutamine, and proliferated at 37°C in an environment comprising of 5% CO2. Cell line authentication was performed using short tandem repeat (STR) fingerprinting.

**Cell Counting Kit-8 (CCK8) assay**

The breast cancer cell lines MCF-7, HCC1599, SK-BR-3, MDA-MB-231, BT-549, ZR-75-1, and 4T1 were obtained from ATCC. All cells were cultured according to standard protocols. Hydrocortisone, insulin, penicillin/streptomycin, HEPES, and L-glutamine were added to enhance the growth of all cell lines, which were proliferated at 37°C in a 5% CO2 environment. Cell line authentication was performed using short tandem repeat (STR) fingerprinting.

**Colony formation assays**

The cells were seeded at 600 cells/well in 6-well plates and cultured for two weeks until visible colonies formed. Colonies were then washed with PBS, fixed with 10% methanol for 30 minutes, and stained with 0.4% crystal violet in 20% ethanol for 20 minutes. After washing and air drying the plates, colony counts were obtained and standard deviations were calculated from three independent experiments.

**Anchorage-independent growth assays**

The assays were conducted using a two-layered solid medium in 6-well plates. We prepared 2% melting Nobel agar and mixed it with DMEM to obtain 0.4% and 0.8% agar, both of which were maintained at 50°C. The 0.8% agar layer was added to the bottom, followed by adding the 0.4% agar suspended with 1 x 10^4^ cells on top. Complete DMEM medium (500 μl) was added to the top layer to maintain its moisture. After 4-6 weeks, the cellular colonies were observed and counted. Standard deviation was calculated based on data from three independent experiments.

**Transwell assay**

To conduct the Transwell assay, we utilized a 24-well Boyden chamber (Cat# 3422, Costar, NY, USA). In the upper chamber, we plated 1 x 10^4^ cells suspended in 200 μl of serum-free medium. The lower chambers were supplemented with DMEM containing 10% FBS. The chambers were incubated at 37℃ for 24 hours. Afterward, the cells in the upper chamber were wiped off with cotton swabs, and those that had migrated to the bottom surface of the membrane were fixed with 1% paraformaldehyde, stained using crystal violet, and counted in five random fields of view per well. This experiment was independently repeated at least three times, and the migration ratio was normalized against the control group.

**Chemical reagents**

The PRMT5 inhibitor GSK3326595 was purchased from MedChemExpress (HY-101563), and was used at a concentration of 0.5 μM in vitro. The insulin was purchased from Thermo Fisher (41400045) and was used at a concentration of 100 nM. The AKT-specific activator SC79 (HY-18749, MedChemExpress) was used at a concentration of 10 μmol/L in vitro.

**Gene expression profiling and analysis**

Analyses were performed using The Cancer Genome Atlas (TCGA) data and Gene expression Omnibus (GEO) publicly available human breast cancer datasets. The analysis of differential TBL2 mRNA expression between cancer and normal samples, and the visualization of the results were carried out using R4.2.0 and the ggplot2 package. GSEA was performed using GSEA program (<http://www.broadinstitute.org/gsea/>).

**GST Pull-Down Assay**

GST–tagged full-length or deletion mutants of TBL2 protein were purified. Then, 5ug GST or GST–tagged proteins were added into cell lysis overnight at 4ºC, followed by the blocked beads for 3 hours. After removal of nonspecific binding proteins, beads were eluted with 2 × SDS loading buffer. Proteins were examined by SDS–polyacrylamide gel electrophoresis and Western blot.

**Supplementary Tables**

**Table S1. Clinicopathological characteristics of 200 breast cancer patients**

| **Parameters** | | | **Number of cases (%)** | |
| --- | --- | --- | --- | --- |
| **Age** | | |  | |
| < 45 | | | 76 (38.0) | |
| ≥ 45 | | | 124 (62.0) | |
| **Clinical stage** | | |  | |
| I-II | | | 99 (49.5) | |
| III-IV | | | 101 (50.5) | |
| **T classification** | | |  | |
| T1-2 | | | 162 (81.0) | |
| T3-4 | | | 38 (19.0) | |
| **N classification** | | |  | |
| N0 | | | 110 (55.0) | |
| N1-N3 | | | 90 (45.0) | |
| **M classification** | | |  | |
| M0 | | | 194 (97.0) | |
| M1 | | | 6 (3.0) | |
| **Histologic grade** | | |  | |
| G1-2 | | | 114 (57.0) | |
| G3 | | | 86 (43.0) | |
| **ER status** | | |  | |
| Positive | | | 109 (54.5) | |
| Negative | | | 91 (45.5) | |
| **PR status** | | |  | |
| Positive | | | 78 (39.0) | |
| Negative | | | 122 (61.0) | |
| **HER2 status** | | |  | |
| Positive | 47 (23.5) | |  |  |
| Negative | | | 153 (76.5) | |
| **Ki-67**  ≥ 15% | | | 113 (56.5) | |
| < 15% | | | 87 (43.5) | |
| **Vital status** | | |  | |
| alive | | | 178 (89.0) | |
| dead | | | 22 (11.0) | |
| **Relapse** | | |  | |
| No | | | 162 (81.0) | |
| Yes | | | 38 (19.0) | |
| **TBL2 expression** | | |  | |
| Low | | | 136 (68.0) | |
| High | | | 64 (32.0) | |

**Table S2. Correlation between TBL2 and clinicopathological characteristics of breast cancer patients**

|  | **TBL2 expression** | |  |
| --- | --- | --- | --- |
| Characteristics | Low,  no. cases (%) | High,  no. cases (%) | *P* values |
| **Age**  < 45  ≥ 45 | 56 (28.0%)  80 (40.0%) | 20 (10.0%)  44 (22.0%) | 0.177 |
| **Clinical stage** |  |  |  |
| I-II | 65 (32.5%) | 34 (17.0%) | 0.482 |
| III-IV | 71(35.5.%) | 30 (15.0%) |  |
| **T** **stage** |  |  |  |
| T1-2 | 116 (58.0%) | 46 (23.0%) | 0.024 |
| T3-4 | 20 (10.0%) | 18 (9.0%) |  |
| **N stage** |  |  |  |
| N0 | 75 (37.5%) | 35 (17.5%) | 0.951 |
| N1-3 | 61 (30.5%) | 29 (14.5%) |  |
| **M stage** |  |  |  |
| M0 | 133 (67.5%) | 61 (29.5%) | 0.566 |
| M1 | 3 (1.5%) | 3 (1.5%) |  |
| **Histologic grade** |  |  |  |
| G1-2 | 77 (38.5%) | 37 (18.5%) | 0.873 |
| G3 | 59(29.5%) | 27 (13.5%) |  |
| **Ki-67** |  |  |  |
| < 15% | 61(30.5%) | 26 (13.0%) | 0.574 |
| ≥ 15% | 75 (37.5%) | 38 (19.0%) |  |
| **Relapse** |  |  |  |
| No | 119 (59.5%) | 43 (21.5%) | 0.001 |
| Yes | 17 (8.5%) | 21 (10.5%) |  |

**Table S3. Immunoprecipitation-mass spectrometry detected TBL2-interacting proteins**

| **293T-TBL2-Flag** | **MDA-MB-231-TBL2-Flag** | **Intersection** |
| --- | --- | --- |
| \| Q9BQG0\|MBB1A_HUMAN \| \| --- \| \| P09874\|PARP1_HUMAN \| \| P78527\|PRKDC_HUMAN \| \| P39023\|RL3_HUMAN \| \| P19338\|NUCL_HUMAN \| \| P06748\|NPM_HUMAN \| \| O14744\|ANM5_HUMAN \| \| P36578\|RL4_HUMAN \| \| Q08211\|DHX9_HUMAN \| \| P52272\|HNRPM_HUMAN \| \| Q9Y2W1\|TR150_HUMAN \| \| Q9NR30\|DDX21_HUMAN \| \| P09651\|ROA1_HUMAN \| \| Q14684\|RRP1B_HUMAN \| \| Q00839\|HNRPU_HUMAN \| \| P05388\|RLA0_HUMAN \| \| Q9BQA1\|MEP50_HUMAN \| \| P62917\|RL8_HUMAN \| \| P26599\|PTBP1_HUMAN \| \| P62424\|RL7A_HUMAN \| \| O00571\|DDX3X_HUMAN \| \| Q8NC51\|PAIRB_HUMAN \| \| Q9UGP8\|SEC63_HUMAN \| \| Q9BQE3\|TBA1C_HUMAN \| \| P0DMV8\|HS71A_HUMAN \| \| P13010\|XRCC5_HUMAN \| \| O75688\|PPM1B_HUMAN \| \| P27635\|RL10_HUMAN \| \| P78347\|GTF2I_HUMAN \| \| P07437\|TBB5_HUMAN \| \| P67809\|YBOX1_HUMAN \| \| P27694\|RFA1_HUMAN \| \| P23588\|IF4B_HUMAN \| \| P56182\|RRP1_HUMAN \| \| P11142\|HSP7C_HUMAN \| \| Q9BVP2\|GNL3_HUMAN \| \| Q5JTH9\|RRP12_HUMAN \| \| P18124\|RL7_HUMAN \| \| Q12906\|ILF3_HUMAN \| \| Q02878\|RL6_HUMAN \| \| P62263\|RS14_HUMAN \| \| P23246\|SFPQ_HUMAN \| \| P61247\|RS3A_HUMAN \| \| Q9NVP1\|DDX18_HUMAN \| \| P68371\|TBB4B_HUMAN \| \| P05455\|LA_HUMAN \| \| O60506\|HNRPQ_HUMAN \| \| P98175\|RBM10_HUMAN \| \| P04264\|K2C1_HUMAN \| \| Q07065\|CKAP4_HUMAN \| \| P31943\|HNRH1_HUMAN \| \| P62701\|RS4X_HUMAN \| \| Q8ND56\|LS14A_HUMAN \| \| Q9UMS4\|PRP19_HUMAN \| \| P12956\|XRCC6_HUMAN \| \| P23396\|RS3_HUMAN \| \| P46777\|RL5_HUMAN \| \| P60709\|ACTB_HUMAN \| \| P17844\|DDX5_HUMAN \| \| Q9NX58\|LYAR_HUMAN \| \| P18621\|RL17_HUMAN \| \| Q9NYF8\|BCLF1_HUMAN \| \| Q9P035\|HACD3_HUMAN \| \| Q7L2E3\|DHX30_HUMAN \| \| Q14103\|HNRPD_HUMAN \| \| P61313\|RL15_HUMAN \| \| Q9P2E9\|RRBP1_HUMAN \| \| P04350\|TBB4A_HUMAN \| \| P22626\|ROA2_HUMAN \| \| P07814\|SYEP_HUMAN \| \| P32969\|RL9_HUMAN \| \| P41091\|IF2G_HUMAN \| \| P11940\|PABP1_HUMAN \| \| O76021\|RL1D1_HUMAN \| \| P61978\|HNRPK_HUMAN \| \| Q8NE71\|ABCF1_HUMAN \| \| P15880\|RS2_HUMAN \| \| P62906\|RL10A_HUMAN \| \| O95373\|IPO7_HUMAN \| \| P42704\|LPPRC_HUMAN \| \| P52597\|HNRPF_HUMAN \| \| Q12905\|ILF2_HUMAN \| \| Q92841\|DDX17_HUMAN \| \| Q8IWS0\|PHF6_HUMAN \| \| O00567\|NOP56_HUMAN \| \| P35637\|FUS_HUMAN \| \| P04406\|G3P_HUMAN \| \| Q9H0A0\|NAT10_HUMAN \| \| Q9BQ67\|GRWD1_HUMAN \| \| O43143\|DHX15_HUMAN \| \| P68104\|EF1A1_HUMAN \| \| P05198\|IF2A_HUMAN \| \| P46782\|RS5_HUMAN \| \| P60842\|IF4A1_HUMAN \| \| P04844\|RPN2_HUMAN \| \| Q13283\|G3BP1_HUMAN \| \| P13645\|K1C10_HUMAN \| \| P62241\|RS8_HUMAN \| \| Q12797\|ASPH_HUMAN \| \| P08238\|HS90B_HUMAN \| \| P05387\|RLA2_HUMAN \| \| P46087\|NOP2_HUMAN \| \| Q15233\|NONO_HUMAN \| \| O00746\|NDKM_HUMAN \| \| P11387\|TOP1_HUMAN \| \| Q96AG4\|LRC59_HUMAN \| \| P51991\|ROA3_HUMAN \| \| P16989\|YBOX3_HUMAN \| \| Q86UE4\|LYRIC_HUMAN \| \| P08708\|RS17_HUMAN \| \| P17987\|TCPA_HUMAN \| \| Q08J23\|NSUN2_HUMAN \| \| Q8IY81\|SPB1_HUMAN \| \| Q01844\|EWS_HUMAN \| \| Q13724\|MOGS_HUMAN \| \| Q8TDN6\|BRX1_HUMAN \| \| O75533\|SF3B1_HUMAN \| \| Q15208\|STK38_HUMAN \| \| P30050\|RL12_HUMAN \| \| P05386\|RLA1_HUMAN \| \| P62888\|RL30_HUMAN \| \| P19474\|RO52_HUMAN \| \| P61353\|RL27_HUMAN \| \| P10809\|CH60_HUMAN \| \| P22087\|FBRL_HUMAN \| \| Q07020\|RL18_HUMAN \| \| P20042\|IF2B_HUMAN \| \| P26196\|DDX6_HUMAN \| \| P04843\|RPN1_HUMAN \| \| P62081\|RS7_HUMAN \| \| Q9Y2X3\|NOP58_HUMAN \| \| O95793\|STAU1_HUMAN \| \| Q01780\|EXOSX_HUMAN \| \| O43390\|HNRPR_HUMAN \| \| P61254\|RL26_HUMAN \| \| P55795\|HNRH2_HUMAN \| \| Q9BUJ2\|HNRL1_HUMAN \| \| P62753\|RS6_HUMAN \| \| P21333\|FLNA_HUMAN \| \| Q96SB4\|SRPK1_HUMAN \| \| P62277\|RS13_HUMAN \| \| Q7Z2W4\|ZCCHV_HUMAN \| \| Q9P258\|RCC2_HUMAN \| \| Q9BVI4\|NOC4L_HUMAN \| \| Q99459\|CDC5L_HUMAN \| \| P06576\|ATPB_HUMAN \| \| P26368\|U2AF2_HUMAN \| \| P05141\|ADT2_HUMAN \| \| P62244\|RS15A_HUMAN \| \| P62140\|PP1B_HUMAN \| \| Q13310\|PABP4_HUMAN \| \| P40429\|RL13A_HUMAN \| \| P62269\|RS18_HUMAN \| \| P46783\|RS10_HUMAN \| \| Q14974\|IMB1_HUMAN \| \| P62280\|RS11_HUMAN \| \| Q13151\|ROA0_HUMAN \| \| Q9Y3I0\|RTCB_HUMAN \| \| P62910\|RL32_HUMAN \| \| P26373\|RL13_HUMAN \| \| Q07955\|SRSF1_HUMAN \| \| Q16875\|F263_HUMAN \| \| Q9UN86\|G3BP2_HUMAN \| \| O75691\|UTP20_HUMAN \| \| P62913\|RL11_HUMAN \| \| P16402\|H13_HUMAN \| \| P14866\|HNRPL_HUMAN \| \| Q02543\|RL18A_HUMAN \| \| P62979\|RS27A_HUMAN \| \| P35579\|MYH9_HUMAN \| \| P16403\|H12_HUMAN \| \| P10412\|H14_HUMAN \| \| P39019\|RS19_HUMAN \| \| Q14498\|RBM39_HUMAN \| \| Q99729\|ROAA_HUMAN \| \| P12236\|ADT3_HUMAN \| \| P46778\|RL21_HUMAN \| \| P62847\|RS24_HUMAN \| \| O95470\|SGPL1_HUMAN \| \| Q9Y295\|DRG1_HUMAN \| \| P62750\|RL23A_HUMAN \| \| Q92499\|DDX1_HUMAN \| \| Q9H0D6\|XRN2_HUMAN \| \| Q86V81\|THOC4_HUMAN \| \| P08865\|RSSA_HUMAN \| \| P46781\|RS9_HUMAN \| \| Q06830\|PRDX1_HUMAN \| \| P25398\|RS12_HUMAN \| \| P62249\|RS16_HUMAN \| \| Q9BQ39\|DDX50_HUMAN \| \| Q00325\|MPCP_HUMAN \| \| Q86UP2\|KTN1_HUMAN \| \| Q14444\|CAPR1_HUMAN \| \| P62829\|RL23_HUMAN \| \| P46776\|RL27A_HUMAN \| \| P35251\|RFC1_HUMAN \| \| P52292\|IMA1_HUMAN \| \| P84090\|ERH_HUMAN \| \| Q13144\|EI2BE_HUMAN \| \| P41252\|SYIC_HUMAN \| \| Q13435\|SF3B2_HUMAN \| \| Q13610\|PWP1_HUMAN \| \| P43243\|MATR3_HUMAN \| \| P84098\|RL19_HUMAN \| \| Q9H7B2\|RPF2_HUMAN \| \| Q9UNX4\|WDR3_HUMAN \| \| P35908\|K22E_HUMAN \| \| P51116\|FXR2_HUMAN \| \| O00139\|KIF2A_HUMAN \| \| Q15393\|SF3B3_HUMAN \| \| Q12849\|GRSF1_HUMAN \| \| Q96EY1\|DNJA3_HUMAN \| \| P62266\|RS23_HUMAN \| \| P50914\|RL14_HUMAN \| \| P84103\|SRSF3_HUMAN \| \| P49411\|EFTU_HUMAN \| \| Q9BZE4\|GTPB4_HUMAN \| \| Q00577\|PURA_HUMAN \| \| Q9NTJ3\|SMC4_HUMAN \| \| P36542\|ATPG_HUMAN \| \| P26641\|EF1G_HUMAN \| \| Q16629\|SRSF7_HUMAN \| \| Q96PK6\|RBM14_HUMAN \| \| P11021\|BIP_HUMAN \| \| P50402\|EMD_HUMAN \| \| P35249\|RFC4_HUMAN \| \| P62854\|RS26_HUMAN \| \| Q9Y224\|RTRAF_HUMAN \| \| Q15366\|PCBP2_HUMAN \| \| P39748\|FEN1_HUMAN \| \| Q16531\|DDB1_HUMAN \| \| Q9BWF3\|RBM4_HUMAN \| \| P05023\|AT1A1_HUMAN \| \| P42285\|MTREX_HUMAN \| \| Q7Z2T5\|TRM1L_HUMAN \| \| P25705\|ATPA_HUMAN \| \| P46779\|RL28_HUMAN \| \| Q13247\|SRSF6_HUMAN \| \| Q8WU90\|ZC3HF_HUMAN \| \| Q8WWY3\|PRP31_HUMAN \| \| P61513\|RL37A_HUMAN \| \| P53007\|TXTP_HUMAN \| \| Q9NR50\|EI2BG_HUMAN \| \| P62318\|SMD3_HUMAN \| \| P68133\|ACTS_HUMAN \| \| Q9NVI7\|ATD3A_HUMAN \| \| Q9H0S4\|DDX47_HUMAN \| \| P31689\|DNJA1_HUMAN \| \| Q9NXS2\|QPCTL_HUMAN \| \| Q9Y3U8\|RL36_HUMAN \| \| Q9Y2H1\|ST38L_HUMAN \| \| P39656\|OST48_HUMAN \| \| Q9UI10\|EI2BD_HUMAN \| \| P54105\|ICLN_HUMAN \| \| P62899\|RL31_HUMAN \| \| Q07666\|KHDR1_HUMAN \| \| P63244\|RACK1_HUMAN \| \| Q9Y3F4\|STRAP_HUMAN \| \| Q14157\|UBP2L_HUMAN \| \| O14979\|HNRDL_HUMAN \| \| O75934\|SPF27_HUMAN \| \| Q99832\|TCPH_HUMAN \| \| P51114\|FXR1_HUMAN \| \| Q01804\|OTUD4_HUMAN \| \| O60832\|DKC1_HUMAN \| \| Q13868\|EXOS2_HUMAN \| \| Q58FF7\|H90B3_HUMAN \| \| O00411\|RPOM_HUMAN \| \| P38919\|IF4A3_HUMAN \| \| Q5T9A4\|ATD3B_HUMAN \| \| O60884\|DNJA2_HUMAN \| \| Q13243\|SRSF5_HUMAN \| \| P06310\|KV230_HUMAN \| \| P15927\|RFA2_HUMAN \| \| Q01081\|U2AF1_HUMAN \| \| P37108\|SRP14_HUMAN \| \| O00178\|GTPB1_HUMAN \| \| O60841\|IF2P_HUMAN \| \| P18077\|RL35A_HUMAN \| \| P62316\|SMD2_HUMAN \| \| P07910\|HNRPC_HUMAN \| \| Q06265\|EXOS9_HUMAN \| \| O75534\|CSDE1_HUMAN \| \| Q9NPD3\|EXOS4_HUMAN \| \| P62987\|RL40_HUMAN \| \| P45880\|VDAC2_HUMAN \| \| Q92522\|H1X_HUMAN \| \| Q9Y383\|LC7L2_HUMAN \| \| Q2NL82\|TSR1_HUMAN \| \| P62841\|RS15_HUMAN \| \| Q15024\|EXOS7_HUMAN \| \| P48651\|PTSS1_HUMAN \| \| P25205\|MCM3_HUMAN \| \| Q02978\|M2OM_HUMAN \| \| O43684\|BUB3_HUMAN \| \| P42766\|RL35_HUMAN \| \| Q9NQT5\|EXOS3_HUMAN \| \| P07305\|H10_HUMAN \| \| P78362\|SRPK2_HUMAN \| \| P63173\|RL38_HUMAN \| \| P13639\|EF2_HUMAN \| \| Q14739\|LBR_HUMAN \| \| Q96HS1\|PGAM5_HUMAN \| \| P33992\|MCM5_HUMAN \| \| Q9BUA3\|SPNDC_HUMAN \| \| Q7KZI7\|MARK2_HUMAN \| \| Q14011\|CIRBP_HUMAN \| \| Q9Y6M1\|IF2B2_HUMAN \| \| P17480\|UBF1_HUMAN \| \| O43663\|PRC1_HUMAN \| \| Q8NHQ9\|DDX55_HUMAN \| \| P35268\|RL22_HUMAN \| \| P42677\|RS27_HUMAN \| \| P25789\|PSA4_HUMAN \| \| Q6P5R6\|RL22L_HUMAN \| \| Q49A26\|GLYR1_HUMAN \| \| O00541\|PESC_HUMAN \| \| P01876\|IGHA1_HUMAN \| \| Q92552\|RT27_HUMAN \| \| Q969Q0\|RL36L_HUMAN \| \| P19525\|E2AK2_HUMAN \| \| Q5T4S7\|UBR4_HUMAN \| \| Q15365\|PCBP1_HUMAN \| \| P40938\|RFC3_HUMAN \| \| P63104\|1433Z_HUMAN \| \| P55209\|NP1L1_HUMAN \| \| Q9H3K6\|BOLA2_HUMAN \| \| P06733\|ENOA_HUMAN \| \| P82650\|RT22_HUMAN \| \| P22392\|NDKB_HUMAN \| \| Q99848\|EBP2_HUMAN \| \| P27816\|MAP4_HUMAN \| \| Q6PKG0\|LARP1_HUMAN \| \| P62826\|RAN_HUMAN \| \| P14868\|SYDC_HUMAN \| \| Q13242\|SRSF9_HUMAN \| \| O43660\|PLRG1_HUMAN \| \| P15531\|NDKA_HUMAN \| \| P60866\|RS20_HUMAN \| \| P83731\|RL24_HUMAN \| \| P63010\|AP2B1_HUMAN \| \| Q16576\|RBBP7_HUMAN \| \| P38646\|GRP75_HUMAN \| \| O43670\|ZN207_HUMAN \| \| Q9BRJ6\|CG050_HUMAN \| \| P16615\|AT2A2_HUMAN \| \| Q8WY22\|BRI3B_HUMAN \| \| P62314\|SMD1_HUMAN \| \| Q01130\|SRSF2_HUMAN \| \| Q92804\|RBP56_HUMAN \| \| P53621\|COPA_HUMAN \| \| P40937\|RFC5_HUMAN \| \| P33778\|H2B1B_HUMAN \| \| Q96GQ7\|DDX27_HUMAN \| \| P62851\|RS25_HUMAN \| \| O75607\|NPM3_HUMAN \| \| P49770\|EI2BB_HUMAN \| \| Q9Y5A9\|YTHD2_HUMAN \| \| P29692\|EF1D_HUMAN \| \| Q9GZR7\|DDX24_HUMAN \| \| P62995\|TRA2B_HUMAN \| \| Q9NQ29\|LUC7L_HUMAN \| \| P27348\|1433T_HUMAN \| \| Q15717\|ELAV1_HUMAN \| \| Q9HCE1\|MOV10_HUMAN \| \| Q13303\|KCAB2_HUMAN \| \| Q9NZM5\|NOP53_HUMAN \| \| P52732\|KIF11_HUMAN \| \| Q99878\|H2A1J_HUMAN \| \| Q9H0U3\|MAGT1_HUMAN \| \| P14678\|RSMB_HUMAN \| \| P78346\|RPP30_HUMAN \| \| Q71UM5\|RS27L_HUMAN \| \| Q14232\|EI2BA_HUMAN \| \| P47914\|RL29_HUMAN \| \| Q9NZ01\|TECR_HUMAN \| \| Q13823\|NOG2_HUMAN \| \| Q9UG63\|ABCF2_HUMAN \| \| Q96QR8\|PURB_HUMAN \| \| Q9UNQ2\|DIM1_HUMAN \| \| O95232\|LC7L3_HUMAN \| \| P01834\|IGKC_HUMAN \| \| Q5C9Z4\|NOM1_HUMAN \| \| Q9Y3E5\|PTH2_HUMAN \| \| P49458\|SRP09_HUMAN \| \| Q9BTZ2\|DHRS4_HUMAN \| \| P48047\|ATPO_HUMAN \| \| P32119\|PRDX2_HUMAN \| \| Q9BXS6\|NUSAP_HUMAN \| \| Q99575\|POP1_HUMAN \| \| P82930\|RT34_HUMAN \| \| O94874\|UFL1_HUMAN \| \| Q9Y2P8\|RCL1_HUMAN \| \| O75569\|PRKRA_HUMAN \| \| Q92973\|TNPO1_HUMAN \| \| P60891\|PRPS1_HUMAN \| \| P30041\|PRDX6_HUMAN \| \| Q9NQT4\|EXOS5_HUMAN \| \| Q13895\|BYST_HUMAN \| \| Q9NZB2\|F120A_HUMAN \| \| A0A075B6R9\|KVD24_HUMAN \| \| P49207\|RL34_HUMAN \| \| Q15046\|SYK_HUMAN \| \| Q15629\|TRAM1_HUMAN \| \| Q9UQ35\|SRRM2_HUMAN \| \| Q9Y520\|PRC2C_HUMAN \| \| O75907\|DGAT1_HUMAN \| \| O43148\|MCES_HUMAN \| \| Q9Y285\|SYFA_HUMAN \| \| P46821\|MAP1B_HUMAN \| \| P14618\|KPYM_HUMAN \| \| P43307\|SSRA_HUMAN \| \| Q12789\|TF3C1_HUMAN \| \| Q14241\|ELOA1_HUMAN \| \| P60660\|MYL6_HUMAN \| \| Q96B26\|EXOS8_HUMAN \| \| P55084\|ECHB_HUMAN \| \| Q9Y265\|RUVB1_HUMAN \| \| Q15149\|PLEC_HUMAN \| \| Q15020\|SART3_HUMAN \| \| Q14257\|RCN2_HUMAN \| \| Q9NVV4\|PAPD1_HUMAN \| \| P23458\|JAK1_HUMAN \| \| P35749\|MYH11_HUMAN \| \| Q5SSJ5\|HP1B3_HUMAN \| \| Q8TCJ2\|STT3B_HUMAN \| \| Q9UNF1\|MAGD2_HUMAN \| \| P08579\|RU2B_HUMAN \| \| O43324\|MCA3_HUMAN \| \| Q8WXX5\|DNJC9_HUMAN \| \| P49756\|RBM25_HUMAN \| \| P01859\|IGHG2_HUMAN \| \| P62805\|H4_HUMAN \| \| P84077\|ARF1_HUMAN \| \| P62937\|PPIA_HUMAN \| \| P02768\|ALBU_HUMAN \| \| Q14669\|TRIPC_HUMAN \| \| Q9UJV9\|DDX41_HUMAN \| \| O60762\|DPM1_HUMAN \| \| Q9NVU7\|SDA1_HUMAN \| \| P19105\|ML12A_HUMAN \| \| P56134\|ATPK_HUMAN \| \| Q8NCA5\|FA98A_HUMAN \| \| P60468\|SC61B_HUMAN \| \| Q70IA6\|MOB2_HUMAN \| \| Q9NUL3\|STAU2_HUMAN \| \| Q9UBX3\|DIC_HUMAN \| \| Q9Y3D9\|RT23_HUMAN \| \| P62861\|RS30_HUMAN \| \| Q13813\|SPTN1_HUMAN \| \| Q14694\|UBP10_HUMAN \| \| Q8N3C0\|ASCC3_HUMAN \| \| P46013\|KI67_HUMAN \| \| P55036\|PSMD4_HUMAN \| \| P57088\|TMM33_HUMAN \| \| Q9UNL2\|SSRG_HUMAN \| \| P62891\|RL39_HUMAN \| \| Q92974\|ARHG2_HUMAN \| \| Q9UHB9\|SRP68_HUMAN \| \| P42695\|CNDD3_HUMAN \| \| Q14258\|TRI25_HUMAN \| \| A6NHT5\|HMX3_HUMAN \| \| P18085\|ARF4_HUMAN \| \| Q8IWX8\|CHERP_HUMAN \| \| Q969S3\|ZN622_HUMAN \| \| Q3KQU3\|MA7D1_HUMAN \| \| P54136\|SYRC_HUMAN \| \| Q92900\|RENT1_HUMAN \| \| Q9BX40\|LS14B_HUMAN \| \| P01861\|IGHG4_HUMAN \| \| Q52LJ0\|FA98B_HUMAN \| \| P09012\|SNRPA_HUMAN \| \| Q9NUQ6\|SPS2L_HUMAN \| \| O60524\|NEMF_HUMAN \| \| O95816\|BAG2_HUMAN \| \| P33993\|MCM7_HUMAN \| \| Q8NCX0\|CC150_HUMAN \| \| A6NHR9\|SMHD1_HUMAN \| \| Q9UH99\|SUN2_HUMAN \| \| Q9Y5Q8\|TF3C5_HUMAN \| \| Q9ULV4\|COR1C_HUMAN \| \| Q09028\|RBBP4_HUMAN \| \| E9PRG8\|CK098_HUMAN \| \| Q9H7D7\|WDR26_HUMAN \| \| Q9H089\|LSG1_HUMAN \| \| P35250\|RFC2_HUMAN \| \| Q9BXP5\|SRRT_HUMAN \| \| P12004\|PCNA_HUMAN \| \| Q8NC56\|LEMD2_HUMAN \| \| Q96TA2\|YMEL1_HUMAN \| \| Q13547\|HDAC1_HUMAN \| \| Q15392\|DHC24_HUMAN \| \| Q9Y239\|NOD1_HUMAN \| \| Q8TDY2\|RBCC1_HUMAN \| \| Q9H1I8\|ASCC2_HUMAN \| \| O94973\|AP2A2_HUMAN \| \| Q68DE3\|USF3_HUMAN \| \| Q6DKI1\|RL7L_HUMAN \| \| O00458\|IFRD1_HUMAN \| \| Q13148\|TADBP_HUMAN \| \| Q96P11\|NSUN5_HUMAN \| \| P27824\|CALX_HUMAN \| \| O95881\|TXD12_HUMAN \| \| O60264\|SMCA5_HUMAN \| \| Q8IY37\|DHX37_HUMAN \| \| Q99755\|PI51A_HUMAN \| \| Q8N5C6\|SRBD1_HUMAN \| \| O14513\|NCKP5_HUMAN \| \| P51571\|SSRD_HUMAN \| \| Q86WZ0\|HEAT4_HUMAN \| \| Q02880\|TOP2B_HUMAN \| \| Q96GA3\|LTV1_HUMAN \| \| Q9BW19\|KIFC1_HUMAN \| \| Q9Y6J9\|TAF6L_HUMAN \| \| Q9UM00\|TMCO1_HUMAN \| \| Q4G0J3\|LARP7_HUMAN \| \| Q5VW36\|FOCAD_HUMAN \| \| Q14966\|ZN638_HUMAN \| \| Q6Y7W6\|GGYF2_HUMAN \| \| Q12802\|AKP13_HUMAN \| | \| P36578\|RL4_HUMAN \| \| --- \| \| P19338\|NUCL_HUMAN \| \| Q9P2E9\|RRBP1_HUMAN \| \| P05388\|RLA0_HUMAN \| \| P62424\|RL7A_HUMAN \| \| Q07065\|CKAP4_HUMAN \| \| P62701\|RS4X_HUMAN \| \| Q02878\|RL6_HUMAN \| \| O14744\|ANM5_HUMAN \| \| Q9NR30\|DDX21_HUMAN \| \| Q71U36\|TBA1A_HUMAN \| \| P04843\|RPN1_HUMAN \| \| Q02543\|RL18A_HUMAN \| \| P62753\|RS6_HUMAN \| \| P06748\|NPM_HUMAN \| \| Q9BQA1\|MEP50_HUMAN \| \| A0A0A0MRZ7\|KVD26_HUMAN \| \| P11940\|PABP1_HUMAN \| \| P68366\|TBA4A_HUMAN \| \| Q9BQG0\|MBB1A_HUMAN \| \| Q12797\|ASPH_HUMAN \| \| P32969\|RL9_HUMAN \| \| P11388\|TOP2A_HUMAN \| \| P18621\|RL17_HUMAN \| \| P27635\|RL10_HUMAN \| \| P16989\|YBOX3_HUMAN \| \| O95373\|IPO7_HUMAN \| \| Q7L2E3\|DHX30_HUMAN \| \| P61313\|RL15_HUMAN \| \| Q5JTH9\|RRP12_HUMAN \| \| P12236\|ADT3_HUMAN \| \| P46778\|RL21_HUMAN \| \| Q92841\|DDX17_HUMAN \| \| P62750\|RL23A_HUMAN \| \| Q07020\|RL18_HUMAN \| \| O95793\|STAU1_HUMAN \| \| P62244\|RS15A_HUMAN \| \| Q5T9A4\|ATD3B_HUMAN \| \| P62906\|RL10A_HUMAN \| \| P04844\|RPN2_HUMAN \| \| P62266\|RS23_HUMAN \| \| Q9UMS4\|PRP19_HUMAN \| \| P16402\|H13_HUMAN \| \| P62263\|RS14_HUMAN \| \| P10412\|H14_HUMAN \| \| P04350\|TBB4A_HUMAN \| \| Q86UE4\|LYRIC_HUMAN \| \| P61254\|RL26_HUMAN \| \| P55795\|HNRH2_HUMAN \| \| P06310\|KV230_HUMAN \| \| O43143\|DHX15_HUMAN \| \| Q7Z2W4\|ZCCHV_HUMAN \| \| P46821\|MAP1B_HUMAN \| \| Q8TCJ2\|STT3B_HUMAN \| \| P46776\|RL27A_HUMAN \| \| P42704\|LPPRC_HUMAN \| \| P01615\|KVD28_HUMAN \| \| O94851\|MICA2_HUMAN \| \| Q9BVP2\|GNL3_HUMAN \| \| P18077\|RL35A_HUMAN \| \| P62847\|RS24_HUMAN \| \| Q9Y2X3\|NOP58_HUMAN \| \| O00567\|NOP56_HUMAN \| \| Q16875\|F263_HUMAN \| \| O75688\|PPM1B_HUMAN \| \| Q02880\|TOP2B_HUMAN \| \| Q9NVP1\|DDX18_HUMAN \| \| P39656\|OST48_HUMAN \| \| Q12906\|ILF3_HUMAN \| \| Q15208\|STK38_HUMAN \| \| P61513\|RL37A_HUMAN \| \| Q8IY17\|PLPL6_HUMAN \| \| O95470\|SGPL1_HUMAN \| \| Q92974\|ARHG2_HUMAN \| \| P63010\|AP2B1_HUMAN \| \| P48651\|PTSS1_HUMAN \| \| Q969Q0\|RL36L_HUMAN \| \| P56182\|RRP1_HUMAN \| \| Q9BUJ2\|HNRL1_HUMAN \| \| Q14684\|RRP1B_HUMAN \| \| P62841\|RS15_HUMAN \| \| Q96SB4\|SRPK1_HUMAN \| \| Q01804\|OTUD4_HUMAN \| \| Q14669\|TRIPC_HUMAN \| \| A0A075B6R9\|KVD24_HUMAN \| \| O60841\|IF2P_HUMAN \| \| P37108\|SRP14_HUMAN \| \| Q9BQ39\|DDX50_HUMAN \| \| Q00577\|PURA_HUMAN \| \| P83731\|RL24_HUMAN \| \| O75569\|PRKRA_HUMAN \| \| P08779\|K1C16_HUMAN \| \| Q86V81\|THOC4_HUMAN \| \| Q9BWF3\|RBM4_HUMAN \| \| Q92522\|H1X_HUMAN \| \| P08865\|RSSA_HUMAN \| \| Q14258\|TRI25_HUMAN \| \| Q16629\|SRSF7_HUMAN \| \| Q9Y2G8\|DJC16_HUMAN \| \| Q12849\|GRSF1_HUMAN \| \| Q15393\|SF3B3_HUMAN \| \| Q14498\|RBM39_HUMAN \| \| Q8WWY3\|PRP31_HUMAN \| \| P05121\|PAI1_HUMAN \| \| Q9P035\|HACD3_HUMAN \| \| P78362\|SRPK2_HUMAN \| \| Q9Y2W1\|TR150_HUMAN \| \| P42677\|RS27_HUMAN \| \| Q96EY1\|DNJA3_HUMAN \| \| P62314\|SMD1_HUMAN \| \| Q14257\|RCN2_HUMAN \| \| Q01780\|EXOSX_HUMAN \| \| P47914\|RL29_HUMAN \| \| P34931\|HS71L_HUMAN \| \| Q96PK6\|RBM14_HUMAN \| \| Q13895\|BYST_HUMAN \| \| P05198\|IF2A_HUMAN \| \| O95782\|AP2A1_HUMAN \| \| Q9NRW3\|ABC3C_HUMAN \| \| P60866\|RS20_HUMAN \| \| P26038\|MOES_HUMAN \| \| P45880\|VDAC2_HUMAN \| \| Q02241\|KIF23_HUMAN \| \| Q13243\|SRSF5_HUMAN \| \| Q13501\|SQSTM_HUMAN \| \| P31689\|DNJA1_HUMAN \| \| Q9H089\|LSG1_HUMAN \| \| Q8N3C0\|ASCC3_HUMAN \| \| P26368\|U2AF2_HUMAN \| \| P15311\|EZRI_HUMAN \| \| Q8TDN6\|BRX1_HUMAN \| \| P55209\|NP1L1_HUMAN \| \| Q8WXH0\|SYNE2_HUMAN \| \| O94973\|AP2A2_HUMAN \| \| Q99848\|EBP2_HUMAN \| \| Q5SSJ5\|HP1B3_HUMAN \| \| P46940\|IQGA1_HUMAN \| \| Q71UM5\|RS27L_HUMAN \| \| Q96QR8\|PURB_HUMAN \| \| P52292\|IMA1_HUMAN \| \| P49756\|RBM25_HUMAN \| \| P62861\|RS30_HUMAN \| \| Q9H2U1\|DHX36_HUMAN \| \| P49458\|SRP09_HUMAN \| \| Q99985\|SEM3C_HUMAN \| \| Q9UN86\|G3BP2_HUMAN \| \| Q3KQU3\|MA7D1_HUMAN \| \| Q49A26\|GLYR1_HUMAN \| \| O43251\|RFOX2_HUMAN \| \| Q9Y2H1\|ST38L_HUMAN \| \| O60762\|DPM1_HUMAN \| \| P40938\|RFC3_HUMAN \| \| Q8IUE6\|H2A2B_HUMAN \| \| P05455\|LA_HUMAN \| \| P49207\|RL34_HUMAN \| \| P53007\|TXTP_HUMAN \| \| Q9UI10\|EI2BD_HUMAN \| \| Q9HCE1\|MOV10_HUMAN \| \| O00560\|SDCB1_HUMAN \| \| Q9UG63\|ABCF2_HUMAN \| \| P63104\|1433Z_HUMAN \| \| Q13242\|SRSF9_HUMAN \| \| P43307\|SSRA_HUMAN \| \| P23458\|JAK1_HUMAN \| \| Q13247\|SRSF6_HUMAN \| \| Q9BQ67\|GRWD1_HUMAN \| \| Q9ULG6\|CCPG1_HUMAN \| \| Q9NQT5\|EXOS3_HUMAN \| \| Q9UGP8\|SEC63_HUMAN \| \| Q16576\|RBBP7_HUMAN \| \| Q9NZ01\|TECR_HUMAN \| \| Q9GZR7\|DDX24_HUMAN \| \| Q5T4S7\|UBR4_HUMAN \| \| P42166\|LAP2A_HUMAN \| \| Q9P258\|RCC2_HUMAN \| \| Q9UBX3\|DIC_HUMAN \| \| Q09028\|RBBP4_HUMAN \| \| Q9NX58\|LYAR_HUMAN \| \| Q9NZM5\|NOP53_HUMAN \| \| P42695\|CNDD3_HUMAN \| \| Q8IY37\|DHX37_HUMAN \| \| P07237\|PDIA1_HUMAN \| \| Q9NPD3\|EXOS4_HUMAN \| \| O75691\|UTP20_HUMAN \| \| O94874\|UFL1_HUMAN \| \| Q5C9Z4\|NOM1_HUMAN \| \| Q9NQ29\|LUC7L_HUMAN \| \| Q16666\|IF16_HUMAN \| \| Q9BX40\|LS14B_HUMAN \| \| Q01081\|U2AF1_HUMAN \| \| P35251\|RFC1_HUMAN \| \| P01859\|IGHG2_HUMAN \| \| Q12789\|TF3C1_HUMAN \| \| A6NHR9\|SMHD1_HUMAN \| \| Q96B26\|EXOS8_HUMAN \| \| Q9UHB9\|SRP68_HUMAN \| \| Q96CW1\|AP2M1_HUMAN \| \| Q8NHQ9\|DDX55_HUMAN \| \| Q9UM00\|TMCO1_HUMAN \| \| P51116\|FXR2_HUMAN \| \| O60884\|DNJA2_HUMAN \| \| Q14974\|IMB1_HUMAN \| \| Q7Z2T5\|TRM1L_HUMAN \| \| O94905\|ERLN2_HUMAN \| \| Q9UJV9\|DDX41_HUMAN \| \| Q9H0S4\|DDX47_HUMAN \| \| Q6P3W7\|SCYL2_HUMAN \| \| Q9BVI4\|NOC4L_HUMAN \| \| P46013\|KI67_HUMAN \| \| Q8WXX5\|DNJC9_HUMAN \| \| Q00005\|2ABB_HUMAN \| \| Q9Y281\|COF2_HUMAN \| \| Q9H3K6\|BOLA2_HUMAN \| \| Q15046\|SYK_HUMAN \| \| O43324\|MCA3_HUMAN \| \| P26641\|EF1G_HUMAN \| \| A7E2V4\|ZSWM8_HUMAN \| \| Q9BUN8\|DERL1_HUMAN \| \| O43660\|PLRG1_HUMAN \| \| Q92552\|RT27_HUMAN \| \| Q5UIP0\|RIF1_HUMAN \| \| O75746\|CMC1_HUMAN \| \| O75607\|NPM3_HUMAN \| \| Q9NQT4\|EXOS5_HUMAN \| \| Q9UNX4\|WDR3_HUMAN \| \| Q06265\|EXOS9_HUMAN \| \| Q9NTJ3\|SMC4_HUMAN \| \| O60732\|MAGC1_HUMAN \| \| O00203\|AP3B1_HUMAN \| \| Q9UNL2\|SSRG_HUMAN \| \| O00541\|PESC_HUMAN \| \| Q9UNQ2\|DIM1_HUMAN \| \| O43852\|CALU_HUMAN \| \| P33947\|ERD22_HUMAN \| \| Q9BRJ6\|CG050_HUMAN \| \| Q9Y6M1\|IF2B2_HUMAN \| \| Q9H7D7\|WDR26_HUMAN \| \| P78527\|PRKDC_HUMAN \| \| P54105\|ICLN_HUMAN \| \| Q8IWX8\|CHERP_HUMAN \| \| O43663\|PRC1_HUMAN \| \| P17480\|UBF1_HUMAN \| \| Q96TA2\|YMEL1_HUMAN \| \| P10620\|MGST1_HUMAN \| \| P17987\|TCPA_HUMAN \| \| O60524\|NEMF_HUMAN \| \| Q6P5R6\|RL22L_HUMAN \| \| Q9NVN8\|GNL3L_HUMAN \| \| Q9Y2X9\|ZN281_HUMAN \| \| Q86TG7\|PEG10_HUMAN \| \| Q86XI2\|CNDG2_HUMAN \| \| Q9UBF1\|MAGC2_HUMAN \| \| Q9BZE4\|GTPB4_HUMAN \| \| O43684\|BUB3_HUMAN \| \| Q15020\|SART3_HUMAN \| \| Q92973\|TNPO1_HUMAN \| \| Q9H8M2\|BRD9_HUMAN \| \| O00458\|IFRD1_HUMAN \| \| P35250\|RFC2_HUMAN \| \| P55036\|PSMD4_HUMAN \| \| P14868\|SYDC_HUMAN \| \| Q12894\|IFRD2_HUMAN \| \| P50281\|MMP14_HUMAN \| \| P50416\|CPT1A_HUMAN \| \| O15240\|VGF_HUMAN \| \| Q8N3F8\|MILK1_HUMAN \| \| Q96EK4\|THA11_HUMAN \| \| P39748\|FEN1_HUMAN \| \| Q13637\|RAB32_HUMAN \| \| Q9Y6J9\|TAF6L_HUMAN \| \| Q14011\|CIRBP_HUMAN \| \| Q92743\|HTRA1_HUMAN \| \| Q6NUQ4\|TM214_HUMAN \| \| Q68D10\|SPT2_HUMAN \| \| P60468\|SC61B_HUMAN \| \| Q8WYP5\|ELYS_HUMAN \| \| P27824\|CALX_HUMAN \| \| O60832\|DKC1_HUMAN \| \| Q6ZUX3\|TGRM2_HUMAN \| \| Q96GA3\|LTV1_HUMAN \| \| O75400\|PR40A_HUMAN \| \| O14686\|KMT2D_HUMAN \| \| Q9Y5Q8\|TF3C5_HUMAN \| \| Q5T655\|CFA58_HUMAN \| \| Q9Y239\|NOD1_HUMAN \| \| Q8NB78\|KDM1B_HUMAN \| \| Q96AQ6\|PBIP1_HUMAN \| \| Q96P11\|NSUN5_HUMAN \| \| Q8NDV3\|SMC1B_HUMAN \| \| O14717\|TRDMT_HUMAN \| \| Q9BU23\|LMF2_HUMAN \| \| P06703\|S10A6_HUMAN \| \| P54132\|BLM_HUMAN \| \| Q8WVM0\|TFB1M_HUMAN \| \| Q8N5L8\|RP25L_HUMAN \| \| Q9H6W3\|RIOX1_HUMAN \| \| P30041\|PRDX6_HUMAN \| \| P48595\|SPB10_HUMAN \| \| Q7Z7G0\|TARSH_HUMAN \| \| P19634\|SL9A1_HUMAN \| \| Q7Z418\|KCNKI_HUMAN \| \| Q8WVM7\|STAG1_HUMAN \| \| O15479\|MAGB2_HUMAN \| \| O43379\|WDR62_HUMAN \| \| Q8N2F6\|ARM10_HUMAN \| \| Q7Z5H4\|VN1R5_HUMAN \| | \| P36578\|RL4_HUMAN \| \| --- \| \| P19338\|NUCL_HUMAN \| \| Q9P2E9\|RRBP1_HUMAN \| \| O14744\|ANM5_HUMAN \| \| P62424\|RL7A_HUMAN \| \| Q07065\|CKAP4_HUMAN \| \| P62701\|RS4X_HUMAN \| \| P05388\|RLA0_HUMAN \| \| Q9BQA1\|MEP50_HUMAN \| \| P04843\|RPN1_HUMAN \| \| Q02543\|RL18A_HUMAN \| \| P62753\|RS6_HUMAN \| \| P06748\|NPM_HUMAN \| \| P62906\|RL10A_HUMAN \| \| P11940\|PABP1_HUMAN \| \| Q9BQG0\|MBB1A_HUMAN \| \| Q12797\|ASPH_HUMAN \| \| P32969\|RL9_HUMAN \| \| P18621\|RL17_HUMAN \| \| P27635\|RL10_HUMAN \| \| P16989\|YBOX3_HUMAN \| \| O95373\|IPO7_HUMAN \| \| Q7L2E3\|DHX30_HUMAN \| \| P61313\|RL15_HUMAN \| \| Q5JTH9\|RRP12_HUMAN \| \| P12236\|ADT3_HUMAN \| \| P46778\|RL21_HUMAN \| \| Q92841\|DDX17_HUMAN \| \| P62750\|RL23A_HUMAN \| \| Q07020\|RL18_HUMAN \| \| O95793\|STAU1_HUMAN \| \| P62244\|RS15A_HUMAN \| \| Q5T9A4\|ATD3B_HUMAN \| \| Q02878\|RL6_HUMAN \| \| P04844\|RPN2_HUMAN \| \| P62266\|RS23_HUMAN \| \| Q9UMS4\|PRP19_HUMAN \| \| P16402\|H13_HUMAN \| \| P62263\|RS14_HUMAN \| \| P10412\|H14_HUMAN \| \| P04350\|TBB4A_HUMAN \| \| Q86UE4\|LYRIC_HUMAN \| \| P61254\|RL26_HUMAN \| \| P55795\|HNRH2_HUMAN \| \| P06310\|KV230_HUMAN \| \| O43143\|DHX15_HUMAN \| \| Q7Z2W4\|ZCCHV_HUMAN \| \| P46821\|MAP1B_HUMAN \| \| Q8TCJ2\|STT3B_HUMAN \| \| P46776\|RL27A_HUMAN \| \| P42704\|LPPRC_HUMAN \| \| Q9BVP2\|GNL3_HUMAN \| \| P18077\|RL35A_HUMAN \| \| P62847\|RS24_HUMAN \| \| Q9Y2X3\|NOP58_HUMAN \| \| O00567\|NOP56_HUMAN \| \| Q16875\|F263_HUMAN \| \| O75688\|PPM1B_HUMAN \| \| Q02880\|TOP2B_HUMAN \| \| Q9NVP1\|DDX18_HUMAN \| \| P39656\|OST48_HUMAN \| \| Q12906\|ILF3_HUMAN \| \| Q15208\|STK38_HUMAN \| \| P61513\|RL37A_HUMAN \| \| O95470\|SGPL1_HUMAN \| \| Q92974\|ARHG2_HUMAN \| \| P63010\|AP2B1_HUMAN \| \| P48651\|PTSS1_HUMAN \| \| Q969Q0\|RL36L_HUMAN \| \| P56182\|RRP1_HUMAN \| \| Q9BUJ2\|HNRL1_HUMAN \| \| Q14684\|RRP1B_HUMAN \| \| P62841\|RS15_HUMAN \| \| Q96SB4\|SRPK1_HUMAN \| \| Q01804\|OTUD4_HUMAN \| \| Q14669\|TRIPC_HUMAN \| \| A0A075B6R9\|KVD24_HUM Q02878\|RL6_HUMAN \| \| O60841\|IF2P_HUMAN \| \| P37108\|SRP14_HUMAN \| \| Q9BQ39\|DDX50_HUMAN \| \| Q00577\|PURA_HUMAN \| \| P83731\|RL24_HUMAN \| \| O75569\|PRKRA_HUMAN \| \| Q86V81\|THOC4_HUMAN \| \| Q9BWF3\|RBM4_HUMAN \| \| Q92522\|H1X_HUMAN \| \| P08865\|RSSA_HUMAN \| \| Q14258\|TRI25_HUMAN \| \| Q16629\|SRSF7_HUMAN \| \| Q12849\|GRSF1_HUMAN \| \| Q15393\|SF3B3_HUMAN \| \| Q14498\|RBM39_HUMAN \| \| Q8WWY3\|PRP31_HUMAN \| \| Q9P035\|HACD3_HUMAN \| \| P78362\|SRPK2_HUMAN \| \| Q9Y2W1\|TR150_HUMAN \| \| P42677\|RS27_HUMAN \| \| Q96EY1\|DNJA3_HUMAN \| \| P62314\|SMD1_HUMAN \| \| Q14257\|RCN2_HUMAN \| \| Q01780\|EXOSX_HUMAN \| \| P47914\|RL29_HUMAN \| \| Q96PK6\|RBM14_HUMAN \| \| Q13895\|BYST_HUMAN \| \| P05198\|IF2A_HUMAN \| \| P60866\|RS20_HUMAN \| \| P45880\|VDAC2_HUMAN \| \| Q13243\|SRSF5_HUMAN \| \| P31689\|DNJA1_HUMAN \| \| Q9H089\|LSG1_HUMAN \| \| Q8N3C0\|ASCC3_HUMAN \| \| P26368\|U2AF2_HUMAN \| \| Q8TDN6\|BRX1_HUMAN \| \| P55209\|NP1L1_HUMAN \| \| O94973\|AP2A2_HUMAN \| \| Q99848\|EBP2_HUMAN \| \| Q5SSJ5\|HP1B3_HUMAN \| \| Q71UM5\|RS27L_HUMAN \| \| Q96QR8\|PURB_HUMAN \| \| P52292\|IMA1_HUMAN \| \| P49756\|RBM25_HUMAN \| \| P62861\|RS30_HUMAN \| \| P49458\|SRP09_HUMAN \| \| Q9UN86\|G3BP2_HUMAN \| \| Q3KQU3\|MA7D1_HUMAN \| \| Q49A26\|GLYR1_HUMAN \| \| Q9Y2H1\|ST38L_HUMAN \| \| O60762\|DPM1_HUMAN \| \| P40938\|RFC3_HUMAN \| \| P05455\|LA_HUMAN \| \| P49207\|RL34_HUMAN \| \| P53007\|TXTP_HUMAN \| \| Q9UI10\|EI2BD_HUMAN \| \| Q9HCE1\|MOV10_HUMAN \| \| Q9UG63\|ABCF2_HUMAN \| \| P63104\|1433Z_HUMAN \| \| Q13242\|SRSF9_HUMAN \| \| P43307\|SSRA_HUMAN \| \| P23458\|JAK1_HUMAN \| \| Q13247\|SRSF6_HUMAN \| \| Q9BQ67\|GRWD1_HUMAN \| \| Q9NQT5\|EXOS3_HUMAN \| \| Q9UGP8\|SEC63_HUMAN \| \| Q16576\|RBBP7_HUMAN \| \| Q9NZ01\|TECR_HUMAN \| \| Q9GZR7\|DDX24_HUMAN \| \| Q5T4S7\|UBR4_HUMAN \| \| Q9P258\|RCC2_HUMAN \| \| Q9UBX3\|DIC_HUMAN \| \| Q09028\|RBBP4_HUMAN \| \| Q9NX58\|LYAR_HUMAN \| \| Q9NZM5\|NOP53_HUMAN \| \| P42695\|CNDD3_HUMAN \| \| Q8IY37\|DHX37_HUMAN \| \| Q9NPD3\|EXOS4_HUMAN \| \| O75691\|UTP20_HUMAN \| \| O94874\|UFL1_HUMAN \| \| Q5C9Z4\|NOM1_HUMAN \| \| Q9NQ29\|LUC7L_HUMAN \| \| Q9BX40\|LS14B_HUMAN \| \| Q01081\|U2AF1_HUMAN \| \| P35251\|RFC1_HUMAN \| \| P01859\|IGHG2_HUMAN \| \| Q12789\|TF3C1_HUMAN \| \| A6NHR9\|SMHD1_HUMAN \| \| Q96B26\|EXOS8_HUMAN \| \| Q9UHB9\|SRP68_HUMAN \| \| Q8NHQ9\|DDX55_HUMAN \| \| Q9UM00\|TMCO1_HUMAN \| \| P51116\|FXR2_HUMAN \| \| O60884\|DNJA2_HUMAN \| \| Q14974\|IMB1_HUMAN \| \| Q7Z2T5\|TRM1L_HUMAN \| \| Q9UJV9\|DDX41_HUMAN \| \| Q9H0S4\|DDX47_HUMAN \| \| Q9BVI4\|NOC4L_HUMAN \| \| P46013\|KI67_HUMAN \| \| Q8WXX5\|DNJC9_HUMAN \| \| Q9H3K6\|BOLA2_HUMAN \| \| Q15046\|SYK_HUMAN \| \| O43324\|MCA3_HUMAN \| \| P26641\|EF1G_HUMAN \| \| O43660\|PLRG1_HUMAN \| \| Q92552\|RT27_HUMAN \| \| O75607\|NPM3_HUMAN \| \| Q9NQT4\|EXOS5_HUMAN \| \| Q9UNX4\|WDR3_HUMAN \| \| Q06265\|EXOS9_HUMAN \| \| Q9NTJ3\|SMC4_HUMAN \| \| Q9UNL2\|SSRG_HUMAN \| \| O00541\|PESC_HUMAN \| \| Q9UNQ2\|DIM1_HUMAN \| \| Q9BRJ6\|CG050_HUMAN \| \| Q9Y6M1\|IF2B2_HUMAN \| \| Q9H7D7\|WDR26_HUMAN \| \| P78527\|PRKDC_HUMAN \| \| P54105\|ICLN_HUMAN \| \| Q8IWX8\|CHERP_HUMAN \| \| O43663\|PRC1_HUMAN \| \| P17480\|UBF1_HUMAN \| \| Q96TA2\|YMEL1_HUMAN \| \| P17987\|TCPA_HUMAN \| \| O60524\|NEMF_HUMAN \| \| Q6P5R6\|RL22L_HUMAN \| \| Q9BZE4\|GTPB4_HUMAN \| \| O43684\|BUB3_HUMAN \| \| Q15020\|SART3_HUMAN \| \| Q92973\|TNPO1_HUMAN \| \| O00458\|IFRD1_HUMAN \| \| P35250\|RFC2_HUMAN \| \| P55036\|PSMD4_HUMAN \| \| P14868\|SYDC_HUMAN \| \| P39748\|FEN1_HUMAN \| \| Q9Y6J9\|TAF6L_HUMAN \| \| Q14011\|CIRBP_HUMAN \| \| P60468\|SC61B_HUMAN \| \| P27824\|CALX_HUMAN \| \| O60832\|DKC1_HUMAN \| \| Q96GA3\|LTV1_HUMAN \| \| Q9Y5Q8\|TF3C5_HUMAN \| \| Q9Y239\|NOD1_HUMAN \| \| Q96P11\|NSUN5_HUMAN \| \| P30041\|PRDX6_HUMAN \| \|  \| |
|  |  |  |
